# Strong yet Flexible TiC-SiC Fibrous Membrane with Long-Time Ultrahigh Temperature Resistance for Sensing in Extreme Environment

**DOI:** 10.1007/s40820-025-02019-1

**Published:** 2026-01-05

**Authors:** Tianyue Yang, Yan Shen, Yangzhong Zhao, Zhongqian Zhao, Xue Zhou, Qianji Chen, Xujing Wang, Yanzi Gou

**Affiliations:** Science and Technology On Advanced Ceramic Fibers and Composites Laboratory, College of Aerospace Science and Engineering, National University of Defense Technology, Changsha, 410073 People’s Republic of China

**Keywords:** TiC-SiC, Fibrous membrane, Flexibility, High temperature stability, Pressure sensing

## Abstract

The demand for sensors capable of operating in extreme environment of the fields, such as aerospace vehicles, aeroengines and fire protection, is rapidly increasing. However, developing flexible ceramic fibrous pressure sensors that combine high temperature stability with robust mechanical properties remains a significant challenge. Herein, through precise multi-scale process control, high-strength (2.1 MPa) TiC-SiC flexible fibrous membrane is successfully fabricated. The membrane exhibits exceptional thermal resistance (2000 °C) and long–term thermal stability (1800 °C for 5 h) in the inert atmosphere. Meanwhile, the TiC-SiC fibrous membrane shows excellent oxidation resistance and still achieves strength of 1.8 MPa after being oxidized at 1200 °C for 1 h in air. Remarkably, TiC-SiC fibrous membrane withstands a load of approximately 1400 times its own weight and the ablation of butane flame (~ 1300 °C) for at least 1 h without breaking. Notably, after heat treatment at 1800 °C for 5 h in an argon atmosphere, the TiC-SiC fibrous membrane even sustains pressure–sensing performance for up to 300 cycles. The membrane exhibits stable resistivity up to 900 °C and shows sensing stability under butane flame. The results of this work provide an effective and feasible solution to fill the research gap of flexible fibrous sensors for extreme environments.

**Supplementary Information:**

The online version contains supplementary material available at 10.1007/s40820-025-02019-1.

## Highlights

- TiC-SiC fibrous membrane exhibits exceptional high–temperature  resistance (2000 °C) and long–term thermal stability (1800 °C for 5 h) in an inert atmosphere.
- TiC-SiC fibrous membrane demonstrates stable resistivity up to 900 °C and shows sensing stability under butane flame (~1300 °C).

**Supplementary Information:**

The online version contains supplementary material available at 10.1007/s40820-025-02019-1.

## Graphical abstract

**Figure:**
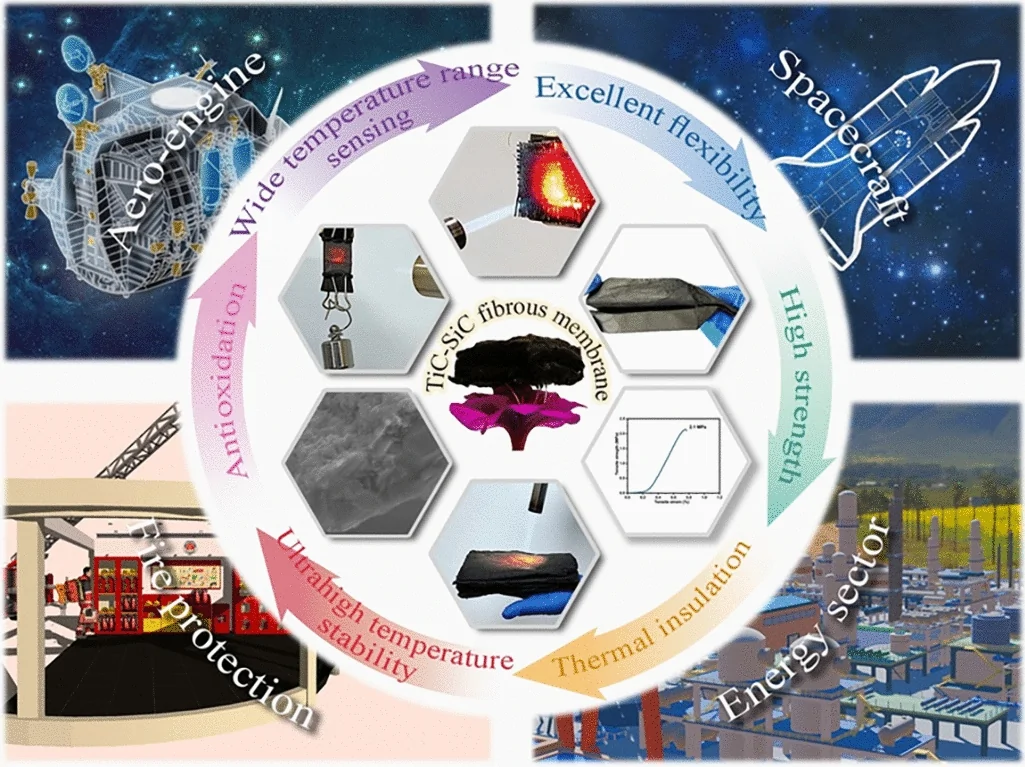

## Introduction

Pressure sensors for extreme environments have significant applications in the fields such as aerospace vehicles, aeroengines and intelligent fire protection [1–7]. For example, sensors for monitoring of aeroengines and aerospace devices need to withstand dynamic deformation, oxidation, and ultrahigh temperatures (~ 2000 °C) [8, 9]. However, traditional sensors are difficult to work stably for a long time in environments of frequent deformation due to their brittleness [10]. While the emerging polymer-based flexible sensors suffer severe performance degradation or even complete failure under high temperature environments [11, 12], MXene–based and carbon materials have been extensively explored as piezoresistive sensors due to their low density, high sensitivity, fast response and superelastic properties [13–17]. Unfortunately, the weak high temperature stability of these sensors hinders their widespread application in oxygen-containing environments. Thereby, there is an urgent need for pressure sensors that can simultaneously achieve good flexibility and excellent thermal stability in various extreme environments [18–20]. Nevertheless, constructing such flexible sensors remains a huge challenge [21–25].

Ceramic ultrafine fibers materials have been considered as an ideal candidate for realizing sensing functions in harsh environments due to their excellent thermal stability and oxidation resistance [26–28]. Chen et al. proposed a silicon oxycarbide ceramic@carbon (SiOC@C) spring-based piezoresistive sensor, realizing good temperature adaptability from –196 to 500 °C [23]. Wei et al. developed a superelastic ZrO_2_–SiO_2_ nanofiber pressure sensor showing stable operation in a wide temperature range from –196 to 800 °C [29]. Although the present ceramic fibrous pressure sensors can defy dynamic deformation [30, 31], the limited high temperature resistance (≤ 800 °C) and poor mechanical properties are still difficult to meet the actual requirement of high temperature application scenarios [32, 33]. Therefore, developing ceramic fibrous pressure sensors with high temperature stability and good mechanical properties is still an impending conundrum in current research [34–36].

In this work, guided by molecular structure design, Ti element was successfully introduced into the precursor of polytitanocarbosilane. Subsequently, TiC was in situ generated through multi-step preparation method, and finally TiC-reinforced SiC (TiC-SiC) fibrous membrane was fabricated. As the second crystalline phase, TiC was randomly distributed in the SiC fibers, which was beneficial for inducing the deflection of cracks, hindering the unstable propagation of cracks, as well as increasing entanglement of filaments, thereby increasing the strength of fibrous membrane up to 2.1 MPa. More importantly, TiC effectively inhibited the abnormal grain growth of SiC in ultrahigh temperature environments, thus improving the thermal stability of the TiC-SiC fibrous membrane up to 2000 °C. Extraordinarily, after being treated at 1800 °C for 5 h in an argon atmosphere, TiC-SiC fibrous membrane still showed good strength and flexibility. Such a long–time high–temperature resistance has not yet been reported for other ultrafine fibers in literature. The membrane can withstand a weight of approximately 1400 times its own weight and remained intact for at least 1 h when being continuously ablated by the butane flame. Notably, the membrane even sustained pressure-sensing performance for up to 300 cycles after heat treatment at 1800 °C for 5 h in an argon atmosphere. Most importantly, the TiC-SiC fibrous membrane exhibited stable resistivity up to 900 °C and showed sensing stability under butane flame. These outstanding performances demonstrated that we had successfully developed a type of novel material that combines long-time ultrahigh-temperature resistance, good mechanical properties as well as high-temperature pressure sensing capabilities, filling the research gap of flexible fibrous sensors for extreme environments.

## Experimental Section

### Materials

Polytitanocarbosilane (PTCS) was synthesized by following the procedure reported in our previous work [37]. By using low-softening-point polycarbosilane (LPCS) and tetrabutyl titanate (Ti(OBu)₄) in different ratios (50:1, 50:3, and 50:5, respectively), the obtained precursor was named as PTCS-1, PTCS-2, and PTCS-3, respectively. Polyvinylpyrrolidone (PVP) was purchased from Aladdin (Shanghai) Co., Ltd. Trichloromethane (CH_3_Cl) was purchased from Sinopharm Chemical Reagent Co., Ltd. It should be noted that all the mentioned chemicals were used as-received without any further purification.

### Fabrication of the TiC-SiC Fibrous Membrane

The spinning solution was prepared by dissolving 1.8 g of PTCS and 0.5 g of PVP in the 10 mL of CH_3_Cl solution, followed by magnetic stirring for approximately 6 h. The obtained homogeneous and clear solution were transferred to syringe pump for electrospinning. The air humidity was 20%. The optimal liquid propulsion speed was 2.0 mL h^−1^, and the optimal spinning voltage was 24 kV. The obtained fibrous membrane was dried in oven at 60 °C for 5 h to remove the excess organic solvent. The obtained PTCS fibrous membrane was cured in air (AC-PTCS) and then pyrolyzed at 1300 °C for 1 h in a nitrogen atmosphere to obtain amorphous Si-Ti-C-O fibrous membrane. The TiC-SiC fibrous membranes were finally obtained after sintering of the Si-Ti-C-O fibrous membrane at 1800 °C for 1 h in an argon atmosphere. As different PTCS-1, PTCS-2, and PTCS-3 polymers were used, the obtained intermediate samples and final product were named as X-1, X-2, and X-3, respectively, where X represented PTCS, AC-PTCS, Si-Ti-C-O and TiC-SiC fibrous membrane, respectively.

### Characterization

The micromorphological analysis was conducted using scanning electron microscopy (SEM, TESCAN MIRA3, Czech Republic) and transmission electron microscopy (TEM, Tecnai F20, USA). The diameter distribution of fibers was quantified by analyzing more than 200 individual fibers according to their SEM images using ImageJ software (Media Cybernetics, USA). Analysis of surface structure and roughness was conducted by using atomic force microscopy (AFM, Bruker Dimension Icon, Germany). Fourier transform infrared spectroscopy (FTIR) was carried out using an infrared spectrometer (Frontier, PerkinElmer, USA). X–ray diffraction (XRD) patterns were recorded on a Bruker AXS D8 Advance diffractometer (Bruker, Germany) equipped with Cu Kα radiation (λ = 1.54178 Å), with 2θ ranging from 10° to 90° at a scanning rate of 10°min⁻^1^. X-ray photoelectron spectroscopy (XPS) analysis was performed using an Escalab 250Xi spectrometer (Thermo Fisher, USA) with an Al Kα excitation source (1487.6 eV). The specific surface area and pore volume were measured by automatic specific surface area and pore volume analyzer (Micromeritics ASAP 2460, USA). Thermal conductivity was measured from the ambient temperature to 1400 °C in an argon atmosphere by the laser thermal conductivity tester (NETZSCH LFA 467, Germany). The density was calculated based on the measured mass and geometric dimensions. The changes of resistance signal and sensing response feature of the fibrous membrane pressure sensor were measured by the flexible electronic array testing system at the ambient temperature (FE–60PT, China). The resistivity of the TiC-SiC fibrous membrane in the temperature range of 25–900 °C was test by variable-temperature resistance testing system (RMS–1200, China). The high-resolution micron-level X-ray computed tomography (X-CT) was used to analyze 3D morphology (EasyTom 160 Micro, France). The mechanical properties of the TiC-SiC fibrous membrane were quantified by Testometric Micro 350 tensile tester. The gauge length and width of the membrane specimens were 25 and 3 mm, respectively. The loading rate was 1 mm min^−1^. The thickness was tested by using a digital fabric thickness gauge (YG141D, China). The stress (*σ*) was calculated by the following equation:* σ* = F/(W × D), where the F, W and D are the load, width and thickness of the fibrous membrane, respectively.

## Results and Discussion

### Fabrication of the Flexible TiC-SiC Fibrous Membrane

**Fig. 1:**
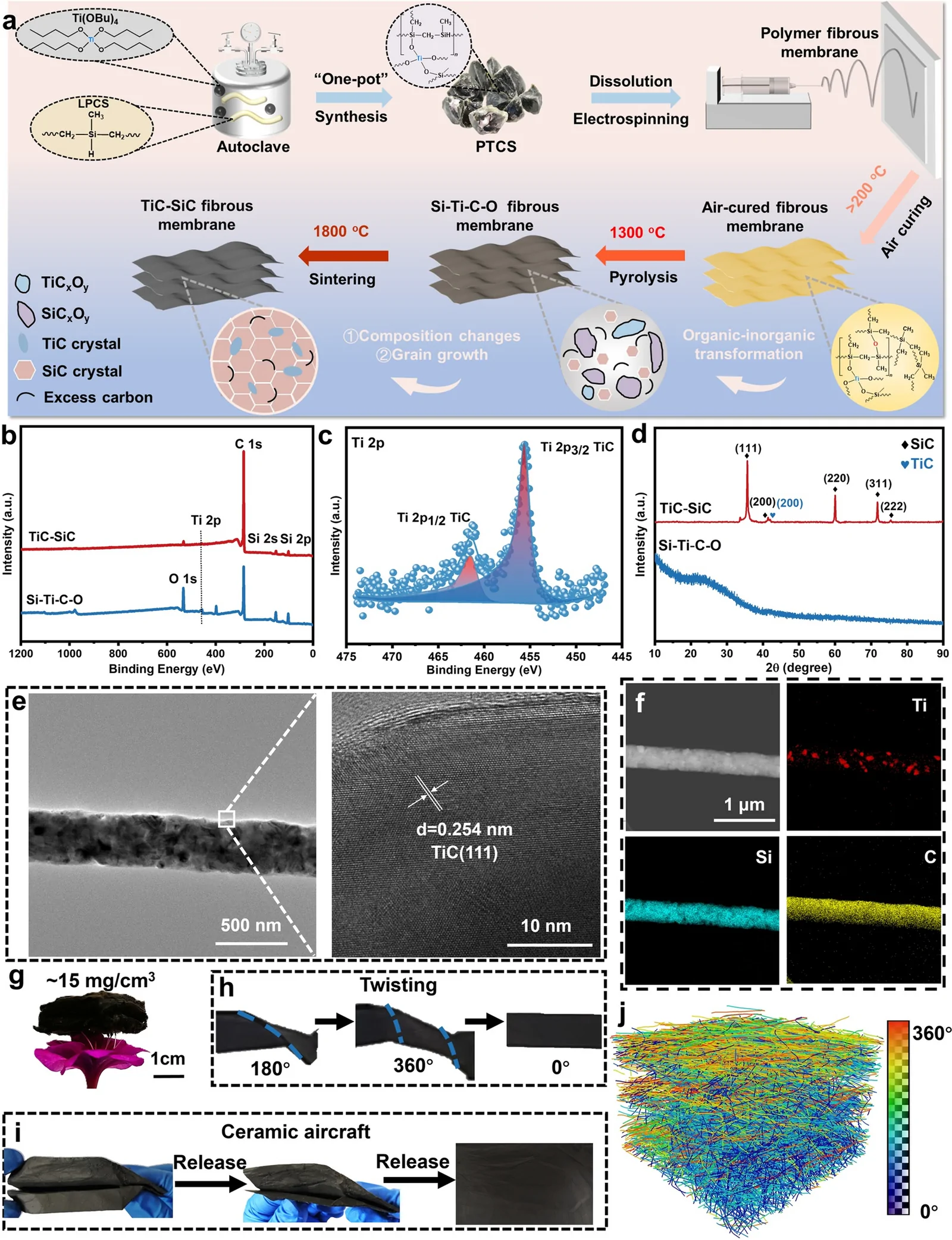
Fabrication of the flexible TiC-SiC fibrous membrane. **a** Schematic of the fabrication process for the TiC-SiC fibrous membrane. **b** XPS spectra and **c** Ti 2*p* spectra of the TiC-SiC fibrous membrane. **d** XRD pattern of the TiC-SiC and Si-Ti-C-O fibrous membranes. **e** TEM and HRTEM images of the TiC-SiC fibrous membrane. **f** Elemental mapping images of the TiC-SiC fiber in the membrane. **g** Optical image of the TiC-SiC fibrous membrane standing on the tip of a flower. **h** Photographs of the TiC-SiC fibrous membrane during the twisting test. **i** Photographs of the TiC-SiC fibrous membrane folded into ceramic aircraft. **j** Three-dimensional (3D) CT image reconstruction of the TiC-SiC fibrous membrane (with different colors indicating the orientation of the fibers). The above demonstrations were all exemplified by the TiC-SiC-3 membrane

As shown by the schematic of the fabrication process (Fig. 1a), PTCS was synthesized firstly by "one–pot" method [37], which was named as PTCS-1, PTCS-2, and PTCS-3, respectively (Figs. S1–S3). Titanium (Ti) contents in these precursors varied from 0.32 to 1.40 wt% (Table S1). The whole fabrication process of the TiC-SiC fibrous membrane involved formation of polymer fibrous membrane, curing, organic–inorganic transformation through pyrolysis and the high–temperature sintering, accompanied by the color changes (Fig. S4). Firstly, by optimizing parameters such as spinning voltage, liquid propulsion speed and spinning humidity, PTCS fibrous membranes formed by fibers with good morphology were prepared by electrospinning method (Figs. S5–S9). Furthermore, air-cured PTCS (AC-PTCS) fibrous membranes were obtained after crosslinking the Si–H bonds of the PTCS green fibers by oxygen in the air (Figs. S10–S11). Subsequently, inorganic Si-Ti-C-O fibrous membranes were fabricated after the AC-PTCS fibrous membranes being pyrolyzed at 1300 °C in a nitrogen atmosphere (Figs. S12–S16 and Table S2). As the high oxygen content (≥ 2.93 wt%) of the Si-Ti-C-O fibrous membrane  was detrimental to their high temperature stability (Table S3), sintering of the membranes at 1800 °C was implemented to eliminate oxygen and obtain the final TiC-SiC fibrous membranes (Figs. S17–S20).

It could be seen from Table S4 that the sintering process effectively reduced the oxygen content to less than 0.39 wt%. Furthermore, Ti contents of the final TiC-SiC fibrous membranes were regulated from 0.50 wt% for TiC-SiC-1 to 1.95 wt% for TiC-SiC-3. This was further confirmed by the results of XPS, which indicated that the main constituent elements were silicon, carbon, oxygen, and titanium. However, the intensity of the O 1*s* peak locating at ~ 532.7 eV descended noticeably for the TiC-SiC fibrous membrane, comparing with that of the Si-Ti-C-O fibrous membrane (Fig. 1b). For the Ti 2*p* spectrum of the TiC-SiC fibrous membrane, two obvious peaks at 461.3 and 455.5 eV were observed ascribing to the Ti–C bonds, and another two peaks at 456.6 and 462.5 eV were assigned to the satellite peaks of Ti–C bonds (Fig. 1c) [38–41]. Moreover, the C 1*s* spectrum further verified the existence of TiC in the fibers (Fig. S21). The patterns of XRD revealed that there appeared five diffraction peaks at 35.4°, 41.3°, 59.8°, 71.5°, and 75.2° for the TiC-SiC fibrous membrane, corresponding to the (111), (200), (220), (311), and (222) crystal faces of *β*–SiC, respectively (Fig. 1d). Notably, the two diffraction peaks at 2θ = 41.6° and 60.5° could be attributed to the (200) and (220) crystal faces of TiC, respectively, which are consistent with the results of XPS analysis. Furthermore, differing from amorphous state of the Si-Ti-C-O fibrous membrane, the narrower width and higher intensity of the peaks for TiC-SiC fibrous membrane could be observed, indicating hugely increasing crystallization of the fiber structure. TEM results further revealed the microstructure of the TiC-SiC fibrous membrane. The single fiber of the membrane was composed of both SiC and TiC grains (Figs. 1e and S22). Meanwhile, the EDS results revealed that the Ti element was randomly distributed inside the fiber **(**Fig. 1f**)**.

The TiC-SiC fibrous membrane exhibited light weight characteristics (~ 15 mg cm^−3^) and excellent flexibility. As shown from Fig. 1g, the TiC-SiC fibrous membrane with a thickness of 1 cm could stand on the petals. Especially, after being twisted for 360 degrees, the membrane could still return to the original appearance (Fig. 1h and Movie S1). Ceramic aircraft was also folded easily, and TiC-SiC fibrous membrane reverted to their original state after removal of the external force (Fig. 1i). The outstanding flexibility of the TiC-SiC fibrous membrane could be ascribed to two reasons. One is the deformation of individual fibers in the membrane. The appearance of the single fiber remained intact after being bended without any obvious cracks (Fig. S23). The other is the special 3D architecture composed of individual fibers. The X–CT reconstructed images of the 3D architecture (Figs. 1j and S24) showed that the membrane was actually constituted by layers of randomly distributed curly TiC-SiC fibers. Both the deformation of individual fibers and the slippage between adjacent layers could result in additional degree of freedom, guaranteeing the flexibility of the whole membrane.

### Mechanical Properties

**Fig. 2:**
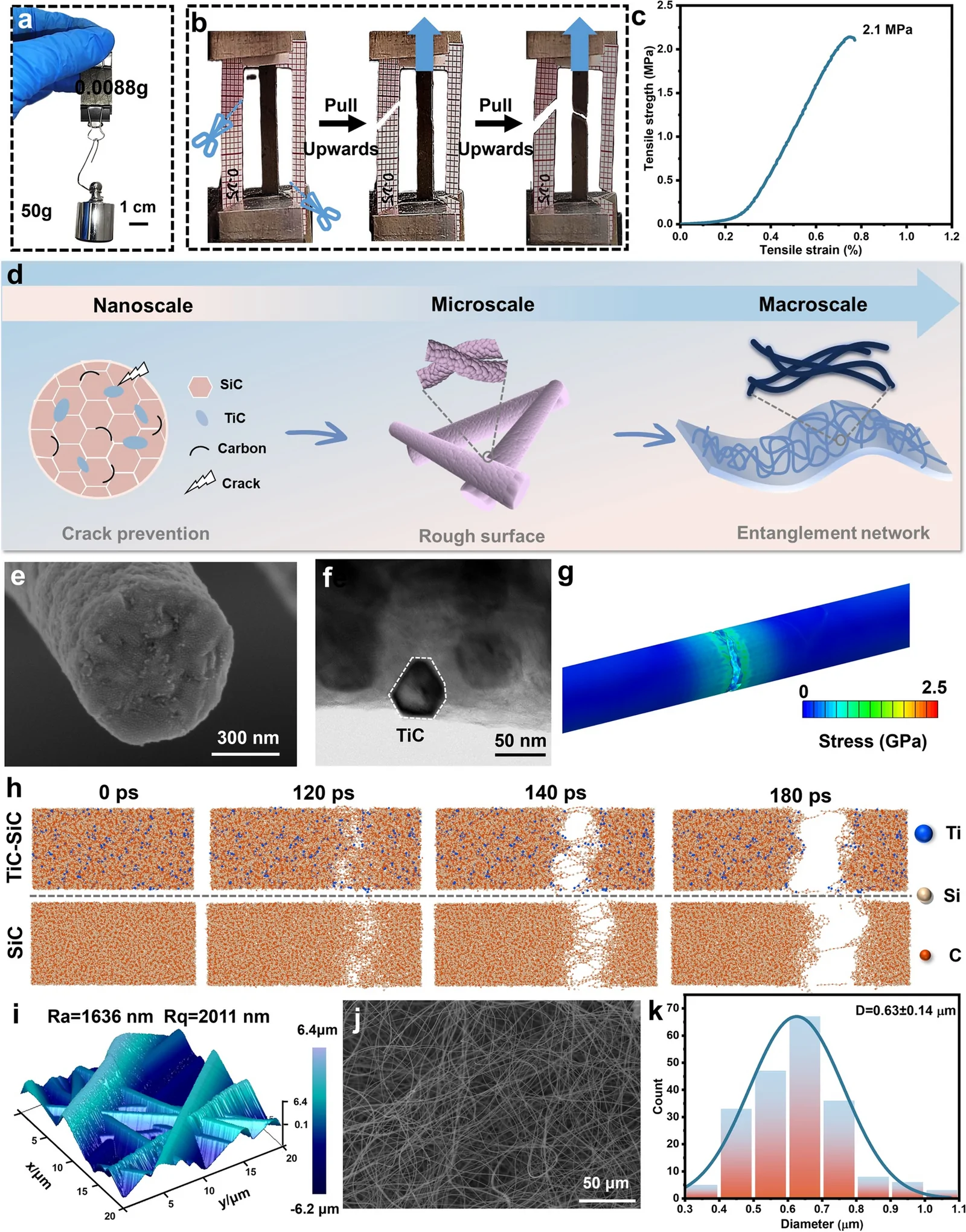
Mechanical properties of the TiC-SiC fibrous membrane. **a** Photograph of the TiC-SiC fibrous membrane bearing a weight of 50 g. **b** Optical photos of mechanical property tests. **c** The stress–strain curve of the TiC-SiC ultrafine fibrous membrane. **d** Schematic diagram of the mechanism of enhanced mechanical properties. **e** The SEM image of the TiC-SiC fibrous membrane. **f** TEM image of the TiC-SiC fiber in the membrane. **g** Finite element analysis of the TiC-SiC fiber. **h** Molecular dynamics simulation during the stretching process. **i** The AFM image of the surface of the TiC-SiC fibrous membrane. **j** The SEM image of the TiC-SiC ultrafine fibers in the membrane.** k** The fiber diameter distribution of the TiC-SiC fibrous membrane

The mechanical performance of the TiC-SiC fibrous membrane was studied in detail, which is essential for its actual application. The TiC-SiC fibrous membrane sample with a width of 1.5 cm, a length of 3 cm and a mass of only 8.8 mg could bear a weight of 50 g (Fig. 2a), indicating excellent load-bearing capacity (more than 5600 times its own weight). The mechanical performance was also tested quantitatively, as shown in Figs. 2b and S25. The maximum tensile strength of the TiC-SiC fibrous membrane was high up to 2.1 MPa (Fig. 2c). For comparison, pure SiC fibrous membrane without titanium element was also prepared (Figs. S26–S28). It is worth noting that the average strength (1.66 MPa) of the TiC-SiC-3 fibrous membrane was the highest among all the membranes (TiC-SiC-1, TiC-SiC-2, and pure SiC fibrous membranes) (Figs. S29–S30).

Although the influencing factors of the strength were complicated, the reasons for such high strength of the TiC-SiC-3 fibrous membrane could be explicated by its distinctive structures in different scale (Fig. 2d). Firstly, the interior nanodefects determine the strength of single fiber, which further affect the strength of the whole membrane. As the sintering step during fabrication process required heterogeneous elements (Ti) as sintering aid to achieve the densification of the fiber [42], there were many pores observed for the pure SiC fiber, as well as the TiC-SiC-1 and TiC-SiC-2 fibers due to inadequate sintering aids (Fig. S31). More densified morphology with reduced internal pores was observed for the TiC-SiC-3 fiber (Fig. 2e and Table S5). TiC grains were also favorable to enhance the mechanical property of the single fiber through mechanisms such as resisting crack propagation and bridging [43]. Based on the "pinning effect" of TiC nanoparticles on cracks, the cracks could be deflected, and the propagation of microcracks could be hindered during the crack propagation process (Figs. 2f and S32). Based on finite element simulation analysis, the fracture of a single fiber was concentrated at the SiC grains rather than TiC, effectively demonstrating the strengthening effect of TiC (Figs. 2g and S33–S35). Furthermore, the results of molecular dynamics simulation also showed that TiC played a patching role. The Ti–C bonds were tightly bonded, and the fracture started from the Si–C bonds, proving that the presence of TiC was conducive to strength improvement of the ceramics (Figs. 2h and S36). In addition, a well-bonded interface will also form between TiC and SiC. This strong interfacial cohesion typically enhances stress tolerance, minimizes interfacial defects, and improves the overall mechanical properties [44, 45]. Secondly, surface roughness of the TiC-SiC-3 fibrous membrane increased significantly (Fig. 2i), comparing with that of pure SiC fibrous membrane (Fig. S37) [46]. The high surface roughness is beneficial to increase friction between adjacent fibers and counteract external tensile force. Additionally, the strength of the TiC-SiC fibrous membrane is also closely related to the distribution state of individual fibers. The TiC-SiC-3 individual fibers are tightly entangled with the smallest diameter reaching approximately 600 nm (Figs. 2j, k and S38–S39**)**, which is advantageous to improvement of the tensile strength of the whole membrane. Based on superior mechanical properties of the TiC-SiC-3 fibrous membrane, it was selected for subsequent investigation of other properties.

### High Temperature Stability

**Fig. 3:**
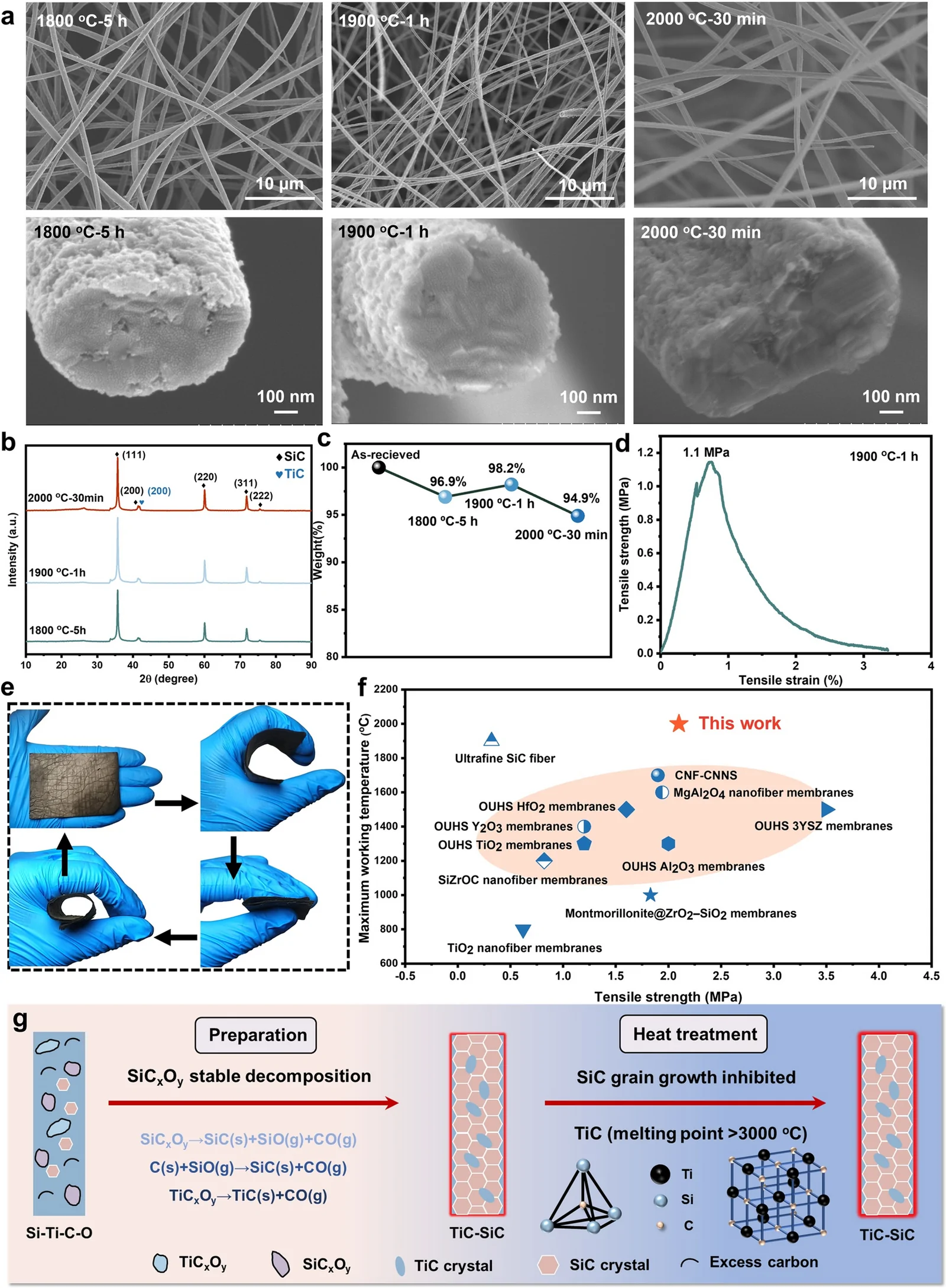
High temperature stability of the TiC-SiC fibrous membrane. **a** SEM images of the TiC-SiC fibrous membranes after heat treatment. **b** XRD patterns of the TiC-SiC fibrous membrane after heat treatment. **c** The weight change of the TiC-SiC fibrous membrane after heat treatment. **d** The stress–strain curve of the TiC-SiC fibrous membrane after heat treatments at 1900 °C for 1 h in an argon atmosphere. **e** Demonstration of the flexibility of the TiC-SiC fibrous membrane after heat treatment at 2000 °C for 30 min. **f** Comparison of tensile strength and the maximum working temperature of different fibrous membranes. **g** Schematic of the high-temperature resistance mechanism of the fibrous membrane

Evaluating the thermal stability is of great significance for the application of high temperature sensors. In this work, the TiC-SiC fibrous membranes were tested at 1800–2000 °C for different times in an argon atmosphere. Surprisingly, the fibrous membranes exhibited excellent stability after being treated at temperature high up to 2000 °C, showing no obvious changes in morphologies of the fiber surface and cross section (Figs. 3a and S40–S45). Especially, the stability of this membrane to withstand high temperature of 1800 °C for the long duration of 5 h is far superior to other fibrous membrane reported in the literature. Furthermore, the XRD patterns confirmed the thermal stability of the crystalline phase of the TiC-SiC fibrous membrane, implying its robustness in ultrahigh temperature environments (Fig. 3b). The weight change of the TiC-SiC fibrous membrane was also recorded (Fig. 3c). It could be seen that after being heated at 1900 °C for 1 h, the weight loss was only 1.8%. Notably, the maximum strength of the TiC-SiC fibrous membrane still remained 1.1 MPa after being treated at 1900 °C for 1 h, demonstrating excellent high temperature resistance of the TiC-SiC fibrous membrane (Fig. 3d). It is noteworthy that all the heat-treated TiC-SiC fibrous membranes also exhibited good flexibility (Figs. 3e and S46–S49), which is favorable to their application in ultrahigh temperature environment. Consequently, TiC-SiC fibrous membrane possesses considerable mechanical strength and the highest working temperature comparing with other ceramic membranes reported in literature (Fig. 3f and Table S6).

The schematic of the high-temperature-resistance mechanism is shown in Fig. 3g. There are multiple factors influencing thermal resistance of the TiC-SiC fibrous membrane. Firstly, when there is a large amount of oxygen in the fibers of the membrane, the resulted amorphous SiC_x_O_y_ phase is unstable, so decomposition reactions as shown in Eqs. (1) and (2) will occur at high temperature [47–49]. This is an important factor causing the abnormal growth of SiC grains during the heat treatment process, ultimately resulting in the collapse of the fiber structure. In this work, the decomposition of the SiC_x_O_y_ phase is regulated in advance during the preparation process, accompanied by elimination of oxygen (from 2.93 to 0.39 wt%). Specifically, the SiC grains can grow to a large size without the pulverization of the fiber structure during the sintering process at 1800 °C. Therefore, for the subsequent heat-treatment process, the thermal driving force is insufficient to cause the secondary growth of the large SiC grains in the fibers of the membrane, which enables the membrane to maintain its original appearance. Furthermore, TiC as ultrahigh temperature phase with a melting point as high as 3000 °C [50] also inhibits the growth of SiC grains during the long–time high–temperature heat treatment and plays a role in stabilizing the SiC matrix (Fig. 2f). Therefore, the unique composition and structure of the fiber membrane endow it with excellent high–temperature resistance.

1 $${\mathrm{SiC}}_{{\mathrm{x}}} {\mathrm{O}}_{{\mathrm{y}}} \left( {\mathrm{s}} \right) \to {\mathrm{SiC}}\left( {\mathrm{s}} \right) + {\mathrm{C}}\left( {\mathrm{s}} \right) + {\mathrm{SiO}}\left( {\mathrm{g}} \right) + {\mathrm{CO}}\left( {\mathrm{g}} \right)$$

2 $${\mathrm{SiO}}\left( {\mathrm{g}} \right) + {\mathrm{2C}}\left( {\mathrm{s}} \right) \, \to {\text{ SiC}}\left( {\mathrm{s}} \right) + {\mathrm{CO}}\left( {\mathrm{g}} \right)$$

### Properties under Integrated Extreme Conditions

**Fig. 4:**
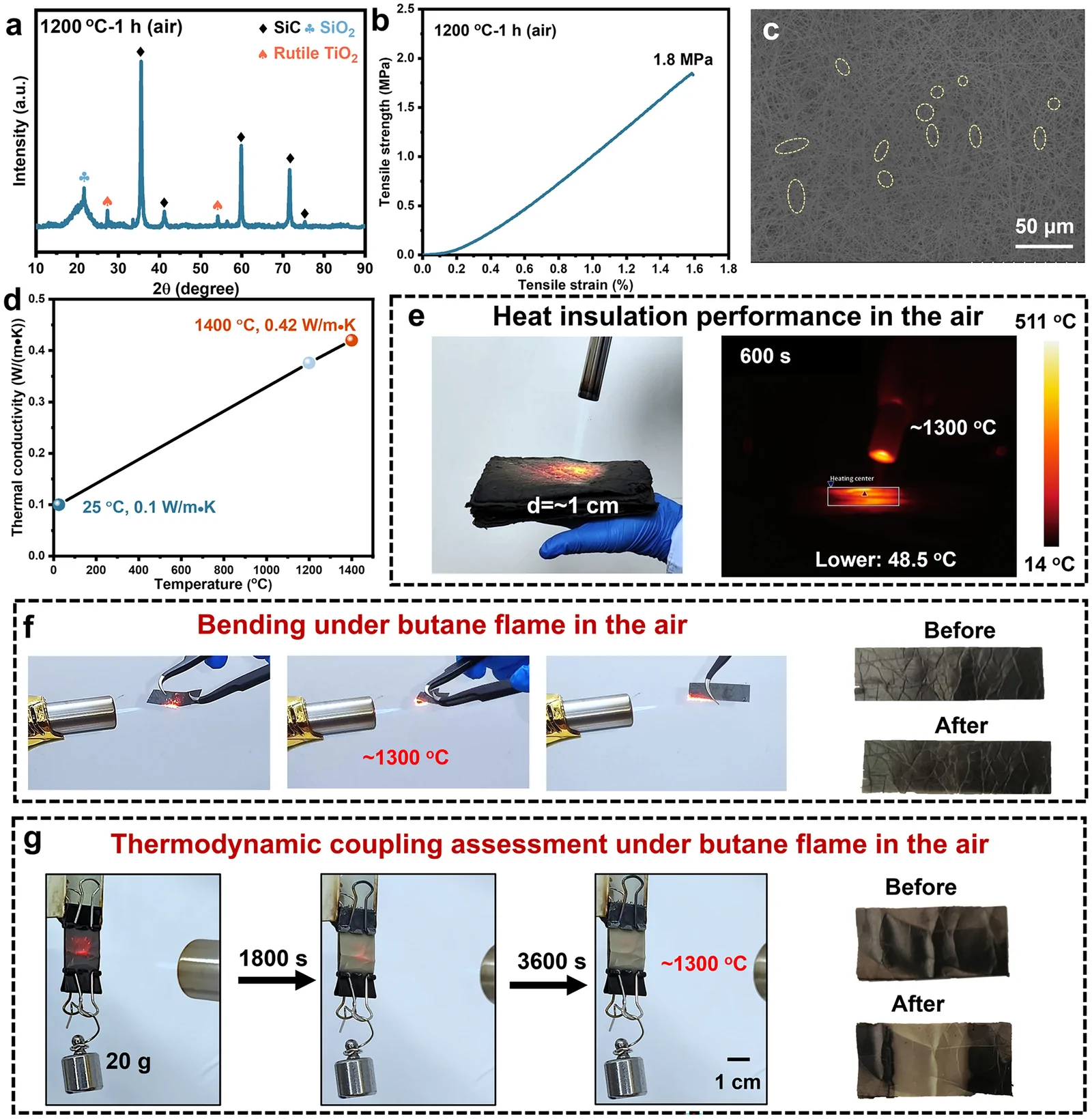
Properties of the TiC-SiC fibrous membrane under integrated extreme conditions. **a** XRD pattern of the TiC-SiC fibrous membrane treated at 1200 °C for 1 h in air. **b** The stress–strain curve of the TiC-SiC ultrafine fibrous membrane after oxidation. **c** The SEM image of the TiC-SiC fibrous membrane after heat treatment at 1200 °C for 1 h in air. **d** Thermal conductivity of the TiC-SiC fibrous membrane at high temperature. **e** Optical and infrared images of the TiC-SiC fibrous membrane being heated by a butane torch. **f** Demonstration of the flexibility of the TiC-SiC fibrous membrane heated with a butane blowlamp. **g** Demonstration of high-temperature-mechanical coupling performance of the TiC-SiC fibrous membrane

To ensure reliable service performance, the TiC-SiC fibrous membrane also needs to possess sufficient stability under integrated extreme conditions. Thereby, the oxidation resistance of the TiC-SiC fibrous membrane was investigated firstly at high temperature in an air atmosphere. XRD pattern shows that after being oxidized at 1200 °C for 1 h, SiO_2_ and TiO_2_ phases appeared in the fiber, while *β*–SiC was still the main component (Fig. 4a). The strength of oxidized TiC-SiC fibrous membrane could still reach 1.8 MPa (Fig. 4b). As shown in Figs. 4c and S50, the fibers of the fibrous membrane were connected at the lap joints due to the formation of the oxide layer on the fiber surface, benefiting for maintaining strength of the membrane. Importantly, after oxidation at 1200 °C for 2 h or at 1400 °C for 1 h, the fibrous membrane still exhibited good mechanical properties (Figs. S51–S56). As the thermal conductivity of the TiC-SiC fibrous membrane was relatively low (0.42 W m^−1^ K^−1^ at 1400 °C) (Fig. 4d), it also exhibited thermal insulation performance at high temperature in air atmosphere, which was demonstrated by heat treatment on the hand using butane flame (~ 1300 °C) (Movie S1). Furthermore, after being heated for 600 s under butane flame, the cold end temperature was less than 63 °C, proving quantitatively the good thermal insulation performance of the TiC-SiC fibrous membrane (Figs. 4e and S57; Movie S1).

Additionally, after being bent under the butane flame, the membranes could still restore its original shape, indicating excellent flexibility (Fig. 4f). Comprehensively, we designed a simple device to demonstrate the mechanical properties of the TiC-SiC fibrous membrane in integrated extreme environment. The TiC-SiC ultrafine fibrous membrane (length: 3 cm, width: 1.5 cm, and weight: 0.0140 g) could remain unbroken for 6000 s pulled by a weight of 20 g (~ 1428 times its own weight) under continuous heating by butane flame, as shown in Figs. 4g and S58; and Movie S1. This further confirmed its outstanding potential to cope with complex conditions in extreme environment.

### Pressure-Sensing Performance

**Fig. 5:**
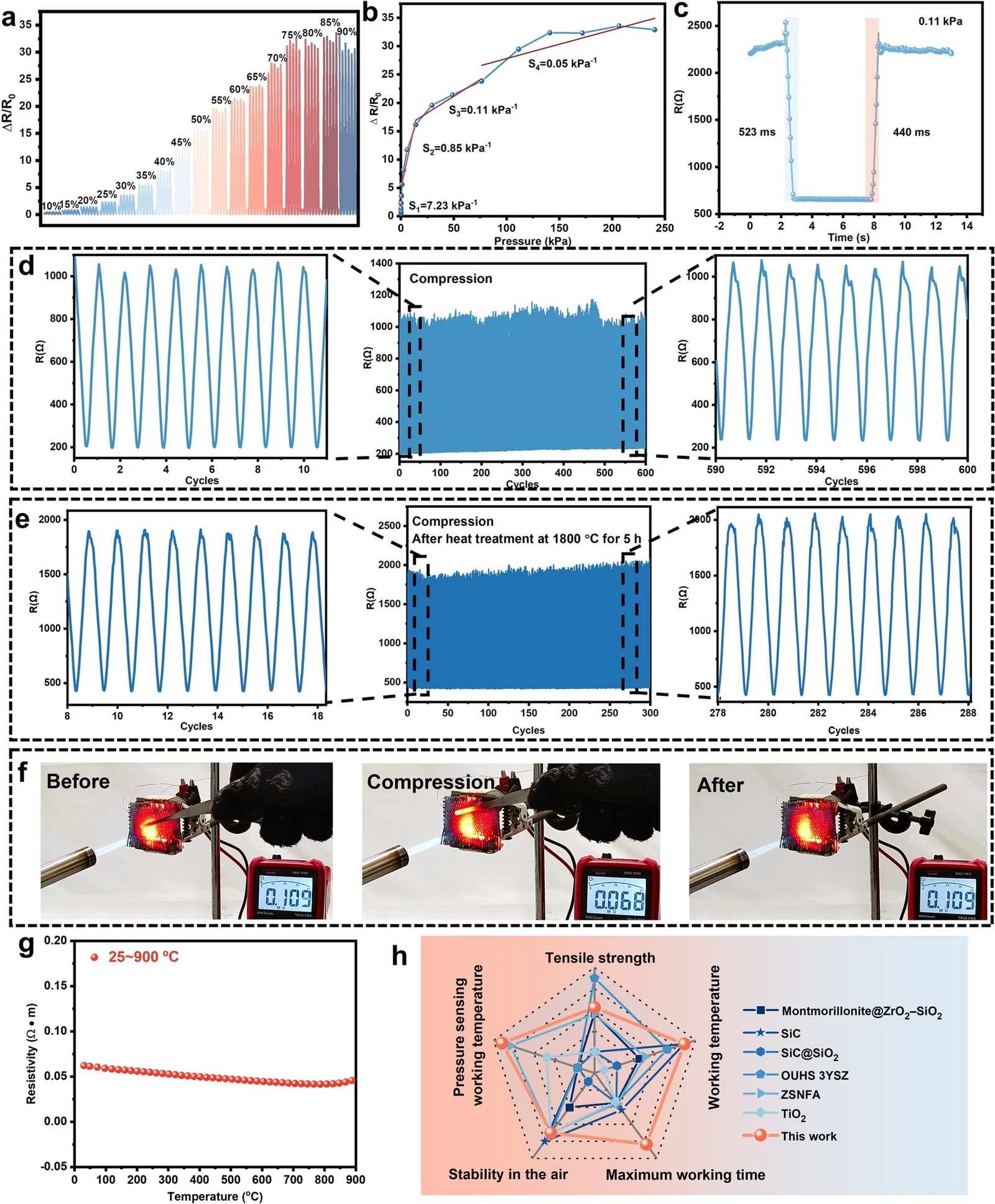
Pressure-sensing performance of the TiC-SiC fibrous membrane. **a** Relative resistance response curves of TiC-SiC fibrous membrane under different strain ranges (10%–90%). **b** The sensitivity of the TiC-SiC fibrous membrane. **c** The response and recovery times of the TiC-SiC fibrous membrane at 0.11 kPa. **d** Electric resistance–cycle responses of the stacked TiC-SiC fibrous membranes under compression and relaxing. **e** Electric resistance–cycle responses of the stacked TiC-SiC fibrous membrane (after heat treatment at 1800 °C for 5 h) under compression and relaxing. **f** Demonstration of high–temperature application of sensor assembled from TiC-SiC fibrous membranes. **g** The resistivity of the TiC-SiC fibrous membrane at different temperature. **h** The radar plot of comparison of the TiC-SiC fibrous membrane in this work with those reported in literature

Based on the good flexibility and mechanical properties of the TiC-SiC fibrous membrane, the pressure–sensing properties of the fibrous membrane were also investigated. As shown in Fig. S59 and Movie S1, the strain of TiC-SiC fibrous membrane was 50%, and the bending angle was approximately 180°. After 600 cycles, the fibers maintained stable electric resistance change (Figs. S60 and S61). In addition, by stacking these fibrous membranes, as shown in Fig. S62 and Movie S1, they could still return to the original shape after release of the compression. The TiC-SiC fibrous membrane exhibited good resistance response signals within the strain range of 10–90% (Fig. 5a). The sensitivity of the TiC-SiC fibrous membrane in the four pressure ranges of 0–0.43, 0.43–14.03, 14.03–76.38, and 76.38–240.25 kPa was 7.23, 0.85, 0.11, and 0.05 kPa^–1^, respectively (Fig. 5b). When the external pressure was 0.11 kPa, the response time of the TiC-SiC fibrous membrane was 523 ms, and the recovery time was 440 ms (Fig. 5c). And after 600 cycles, TiC-SiC fibrous membrane maintained excellent pressure sensing performance, which benefited from the layered stacking structure of the membranes (Figs. 5d and S63). Importantly, TiC-SiC fiber membrane still exhibited excellent resistance response signals after heat treatment at 1800 °C for 5 h in an argon atmosphere, closely related to the high-temperature stability **(**Fig. 5e). Furthermore, after being heated by the butane flame for 60 s, it still exhibited good compression sensing performance (Fig. S64 and Movie S1).

To evaluate the practical sensing performance of the TiC-SiC fibrous membrane, a TiC-SiC fibrous membrane-based high temperature sensor was assembled (Fig. S65). By using two pieces of two–dimensional woven cloth of conventional SiC fibers as the upper and lower surfaces, the fibrous membrane was sandwiched in the middle with copper wire connecting to a digital multimeter. With continuous ablation by the butane flame, the electric resistance of the sensor changed evidently with compression (Fig. 5f and Movie S1). However, it could return to the original value after release of the compression. This could be ascribed to the unchanged resistivity with temperature (25–900 °C) (Fig. 5g). By comparing with the comprehensive performance of the fibrous membrane reported in relevant literature (Fig. 5h and Table S7), it could be seen that the TiC-SiC fibrous membrane in this work has broken the working limits of current fibrous membrane-based pressure sensors. More importantly, unlike other sensors, the TiC-SiC fibrous membrane does not require any substrate for encapsulation, which ensures its excellent pressure-sensing performance without sacrificing the high temperature resistance, making it appealing as a new type of structure–function integrated material.

## Conclusions

In summary, the TiC-SiC fibrous membrane was successfully fabricated based on the molecular design and multi-step preparation. TiC was pinned into the SiC matrix, enhancing the strength of the TiC-SiC fibrous membrane (up to 2.1 MPa). In addition, as an ultra–high temperature phase, TiC could inhibit the abnormal growth of SiC grains under high temperature for a long time. Meanwhile, owing to the extremely low oxygen content (0.39 wt%), the membrane exhibited exceptional thermal resistance (2000 °C) and long–term thermal stability (1800 °C for 5 h) in the inert atmosphere. Importantly, the TiC-SiC fibrous membrane could bear a load of approximately 1400 times its own weight and remained intact after being ablated by the butane flame (~ 1300 °C) for 1 h. Notably, the membrane even sustained pressure–sensing performance for up to 300 cycles after heat treatment at 1800 °C for 5 h in an argon atmosphere. Most importantly, the TiC-SiC fibrous membrane exhibited stable resistivity up to 900 °C and showed sensing stability under butane flame. Thereby, these comprehensive characteristics firmly establish the TiC-SiC fibrous membrane as a transformative solution of high-temperature pressure sensing for extreme environments.

## Supplementary Information

Below is the link to the electronic supplementary material.

Supplementary file1 (DOCX 9255 KB)

Supplementary file2 (MP4 16670 KB)
