# Supplementary material for: Strong yet Flexible TiC-SiC Fibrous Membrane with Long-Time Ultrahigh Temperature Resistance for Sensing in Extreme Environment
Source: Nanomicro Lett. 2026 Jan 5;18:177. doi: 10.1007/s40820-025-02019-1 (PMC12765755; doi:10.1007/s40820-025-02019-1)
Supplement: Supplementary file 1 — Supplementary file1 (DOCX 9255 KB) [file 40820_2025_2019_MOESM1_ESM.docx]

Supporting Information for

**Strong yet Flexible TiC-SiC Fibrous Membrane with Long–Time Ultrahigh Temperature Resistance for Sensing in Extreme Environment**

Tianyue Yang^1^, Yan Shen^1^, Yangzhong Zhao^1^, Zhongqian Zhao^1^, Xue Zhou^1^, Qianji Chen^1^, Xujing Wang^1^, Yanzi Gou^1^*

^1^Science and Technology on Advanced Ceramic Fibers and Composites Laboratory, College of Aerospace Science and Engineering, National University of Defense Technology, Changsha 410073, P. R. China

*Corresponding author. E-mail: y.gou2012@hotmail.com (Yanzi Gou)

**S1 Experimental part**

**S1.1 Elemental composition analysis**

The oxygen content of SiC fibrous membranes was measured by EMGA-820 oxygen and nitrogen analyzer. The carbon content was measured by EMIA-320V2 carbon-sulfur analyzer. The content of silicon element was determined by melting the sample with strong alkali for colorimetric analysis. Analysis of titanium content by Inductively Coupled Plasma Optical Emission Spectrometer (ICP-OES, Thermo ICP 6300).

**S1.2 Mechanical Strength Test**

The mechanical properties of the TiC-SiC fibrous membrane were quantified by Testometric Micro 350 tensile tester. The gauge length and width of the membrane specimens were 25 mm and 3 mm, respectively. The loading rate was 1 mm·min^−1^. The thickness was tested by using a digital fabric thickness gauge (YG141D, China).

The stress (σ) was calculated by the following equation:

σ=F/(W×D)

Where the F, W and D are the load, width and the thickness of the fibrous membrane, respectively.

**S1.3 Finite element analogy**

A finite element analysis software was used, and a microstructure generation algorithm based on Voronoi polygons was adopted to finally establish TiC-SiC composite model. The fiber diameter is 500 nm and the length is 30 μm. Typical uniaxial tensile boundary conditions were used to simulate the overall stress-strain response during the tensile process. The displacement control method was used for loading, and the maximum strain was set to 0.5. A fracture stop criterion was also set: once the principal strain exceeds the critical value, the element will fail, and the simulation will automatically enter the termination process.

**S1.4 Molecular Dynamics Simulations**

Molecular dynamics simulations were performed using LAMMPS 2024 to study the structural and mechanical properties of SiC and TiC-SiC systems. The initial atomic structures were obtained from the crystallographic information file (CIF) of SiC downloaded from a crystallographic database. SiC system: The interatomic interactions were modeled using the Tersoff potential, which accurately describes covalent bonding in SiC. TiC-SiC system: A hybrid potential combining the Tersoff potential for SiC and the Lennard-Jones (LJ) potential for Ti interactions was employed to capture the mixed bonding nature. The parameters for the hybrid potential were combined appropriately within LAMMPS. The system was first equilibrated at room temperature (298 K) using a timestep of 1 fs (0.001 ps). The thermal treatment followed a multi-stage process: Heating under an NPT ensemble (constant number of particles, pressure, and temperature) to 2073 K over 1 ns. Holding at 2073 K under an NVT ensemble (constant number of particles, volume, and temperature) for 1 ns to stabilize the high-temperature phase. Cooling back to 298 K under an NPT ensemble over 1 ns to obtain the relaxed pre-stretch configuration. The equilibrated model was further equilibrated at 298 K under NPT conditions for 1 ns with the same timestep. Subsequent uniaxial tensile deformation was applied at a strain rate of 0.005 Å/ps. Applying the elongation under an NVT ensemble for 200 ps to monitor the mechanical response at constant volume and temperature. Data analysis and visualization of atomic configurations and deformation behavior were conducted using OVITO software, enabling detailed structural interpretation and presentation of results.

**Supplementary Figures and Tables**

**Fig. S1** The synthetic route of PTCS precursor


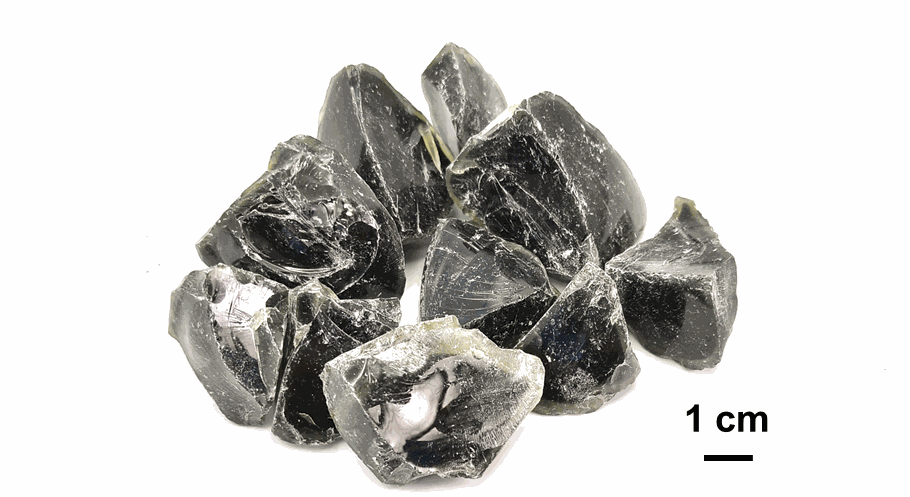


**Fig. S2** The optical photo of PTCS

**Fig. S3** FTIR spectra of the PTCS precursors

**Table S1** The Elemental composition and molecular weight of PTCS precursors

| Sample | Si content (wt%) | C content (wt%) | O content (wt%) | Ti content (wt%) | *M*_w_  (g/mol) | *M*_n_  (g/mol) |
| --- | --- | --- | --- | --- | --- | --- |
| PTCS-1 | 57.8 | 40.33 | 1.50 | 0.32 | 5300 | 1600 |
| PTCS-2 | 57.1 | 40.02 | 2.05 | 0.80 | 7300 | 2100 |
| PTCS-3 | 55.9 | 39.91 | 2.73 | 1.40 | 10700 | 2400 |


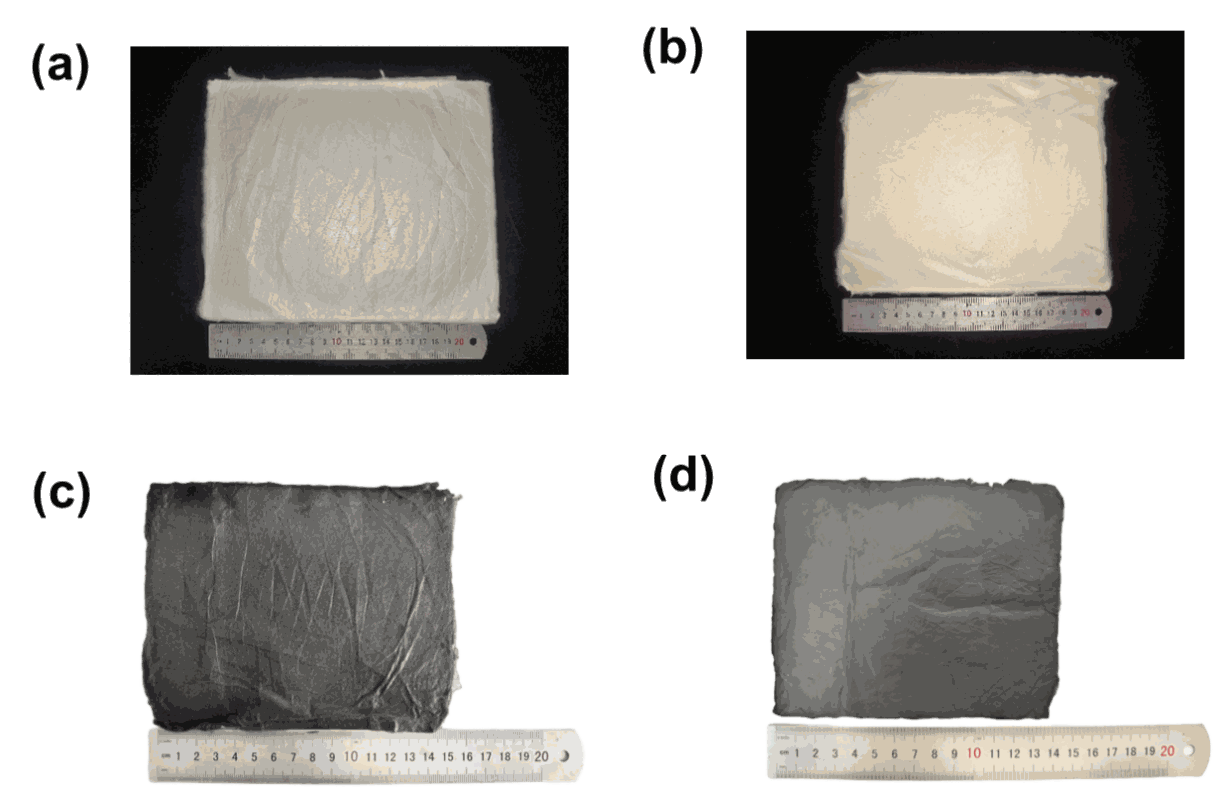


**Fig. S4** Optical photograph of membrane consisted of (**a**) PTCS green fibrous membrane, (**b**) cured fibrous membrane, (**c**) Si-Ti-C-O fibrous membrane, (**d**) TiC-SiC fibrous membrane

The influence of spinning voltage on fiber formation was investigated. When the environmental humidity was 50% and the liquid pushing speed was 2.5 ml/h, three spinning voltage parameters were set as: 16 kV, 20 kV, and 24 kV. At a voltage of 16 kV, the fibers showed a beaded shape, indicating that the fibers were not sufficiently stretched. When the voltage was increased to 20 kV, the fibers still contained spindles. As the voltage increased to 24 kV, the fibers were fully stretched during the spinning process, and the spindles disappeared. When the voltage was further increased, a discharge phenomenon occurred at the needle tip, making spinning impossible. Therefore, 24 kV was finally selected as the optimal spinning voltage.


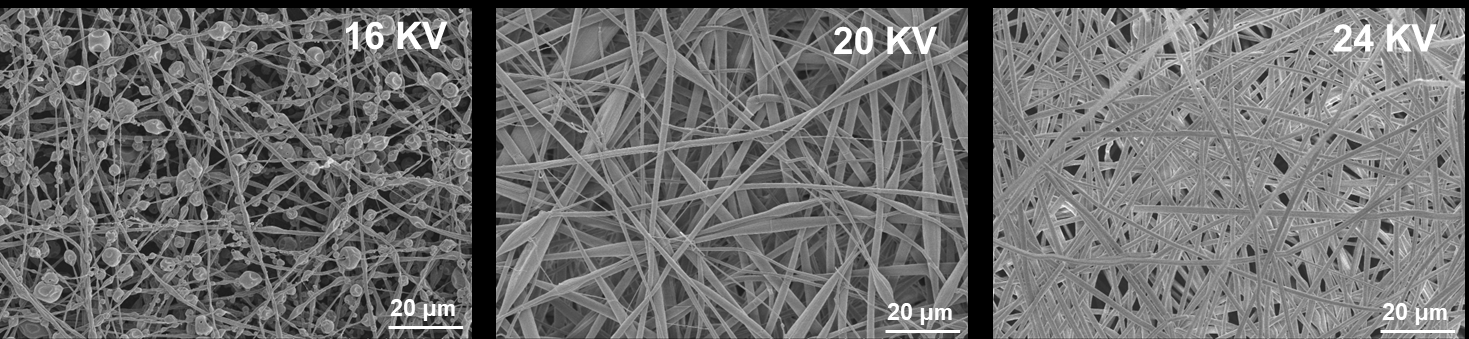


**Fig. S5** SEM images of PTCS fibrous membranes obtained with different spinning voltage

During the electrospinning process, the spinning rate was determined by the syringe pushing speed. When the environmental humidity was 50%, the pushing speed was 2.5 ml/h, and the spinning voltage was 24 kV, three pushing speeds were set as: 1.5 ml/h, 2.0 ml/h, and 2.5 ml/h. The experiment showed that when the solution pushing speed was too low, the speed at which the spinning solution accumulates at the needle tip was lower than the jet ejection speed, resulting in intermittent ejection of the solution at the spinneret, unstable spinning, and discontinuous fibers. When the solution pushing speed increased to 2.0 ml/h, a stable Taylor cone formed at the needle tip, and the jet was fully stretched and refined in the electric field, resulting in fibers with small and uniform diameters. When the solution pushing speed increased to 2.5 ml/h, the ejection became unstable, and the fiber diameters increased and became non-uniform.


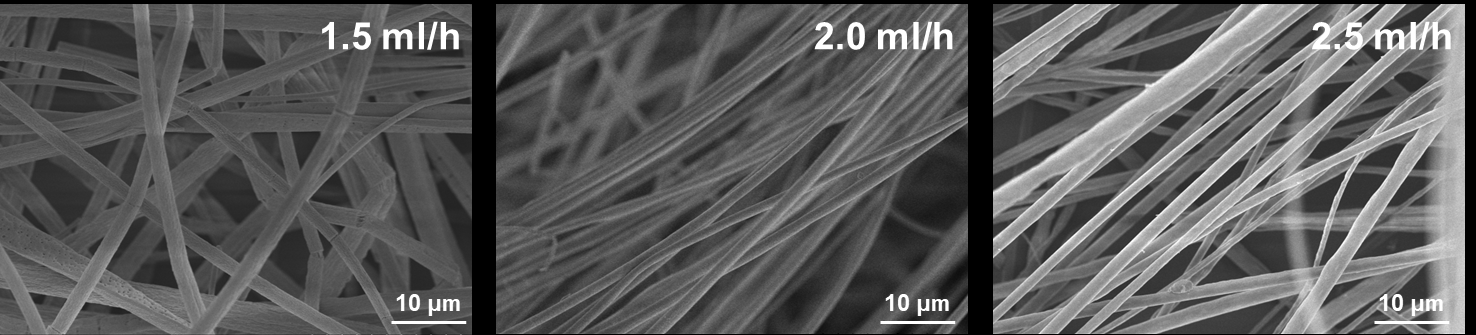


**Fig. S6** SEM images of PTCS fibrous membranes obtained with different pushing speeds

Further regulation was carried out around the spinning humidity, and the humidity was controlled at 20%, 40%, and 60% respectively. When the humidity was 60%, the shape of the fibers was irregular, and a large number of pores appeared on the surface. This is because in a high - humidity environment, the volatilization of the solvent was inhibited, and the surface tension of the spinning solution decreased, resulting in an increase in the fiber diameter. Moreover, as the solvent volatilization took away part of the heat, the temperature of the surface of the polymer solution jet was lower than the ambient temperature. Therefore, small droplets would be deposited on the surface of the jet. After the fibers hardened, pores would be left where the small droplets were located. When the humidity was 40%, the defects on the fiber surface were significantly reduced; when the humidity was reduced to 20%, the fiber surface was smooth with no obvious defects. When the humidity was further reduced, the solution solidified rapidly at the spinneret, causing the spinneret to clog and making normal spinning impossible. Therefore, the spinning experiment was finally carried out with the ambient humidity controlled at 20%.


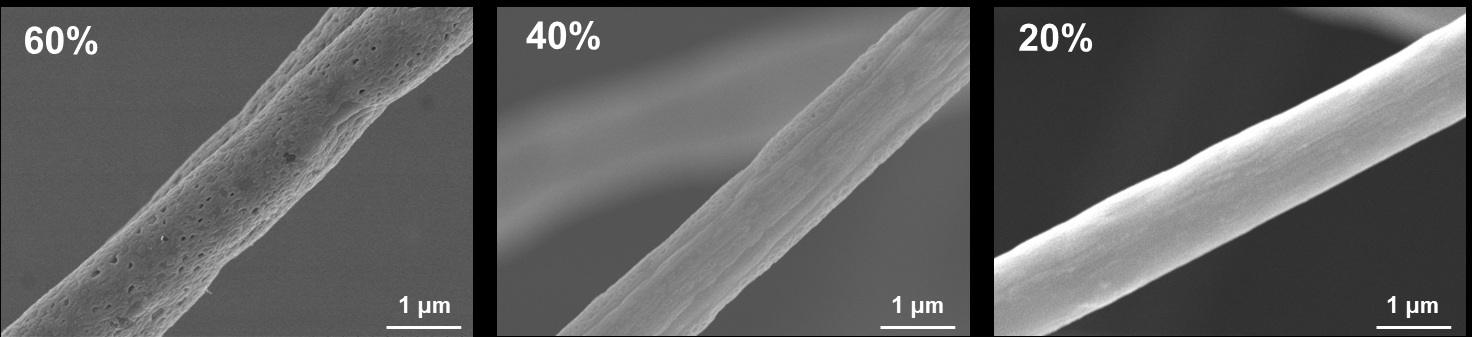


**Fig. S7** SEM images of PTCS fibrous membranes obtained with different humidity


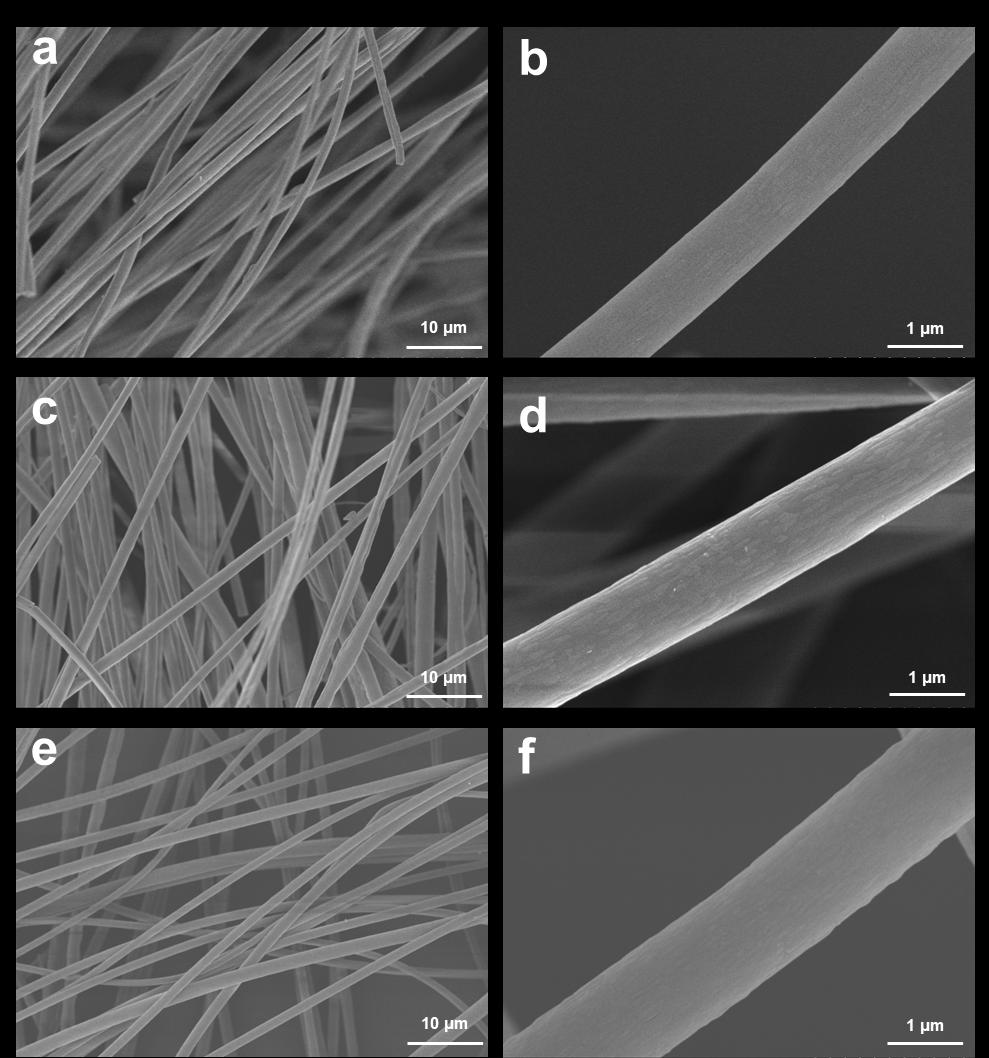


**Fig. S8** The SEM images of PTCS fibrous membranes: (**a-b**) PTCS-1, (**b-c**) PTCS-2, (**c-d**) PTCS-3

**Fig. S9** FTIR spectra of the PTCS fibrous membranes


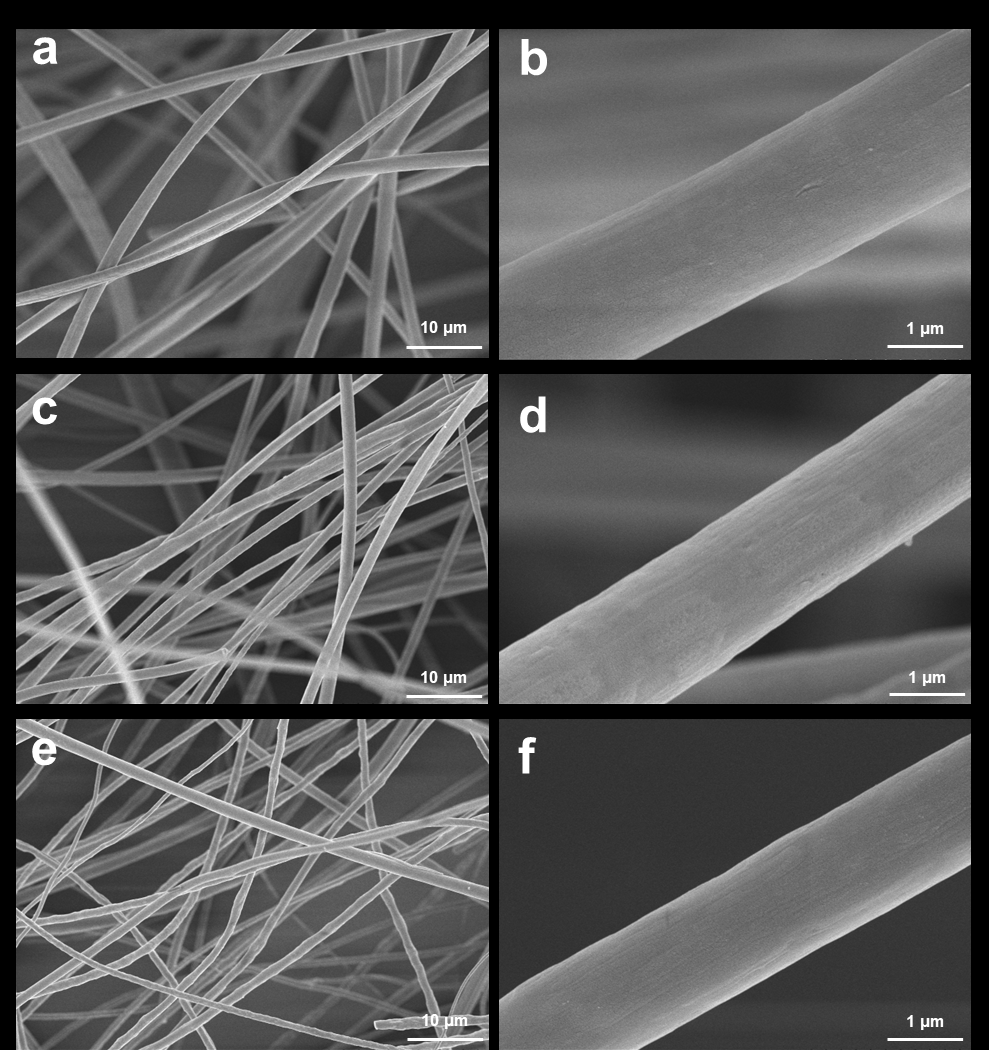


**Fig. S10** The SEM images of AC-PTCS fibrous membranes: (**a-b**) AC-PTCS-1, (**c-d**) AC-PTCS-2, (**e-f**) AC-PTCS-3

**Fig. S11** FTIR spectra of the AC-PTCS fibrous membranes


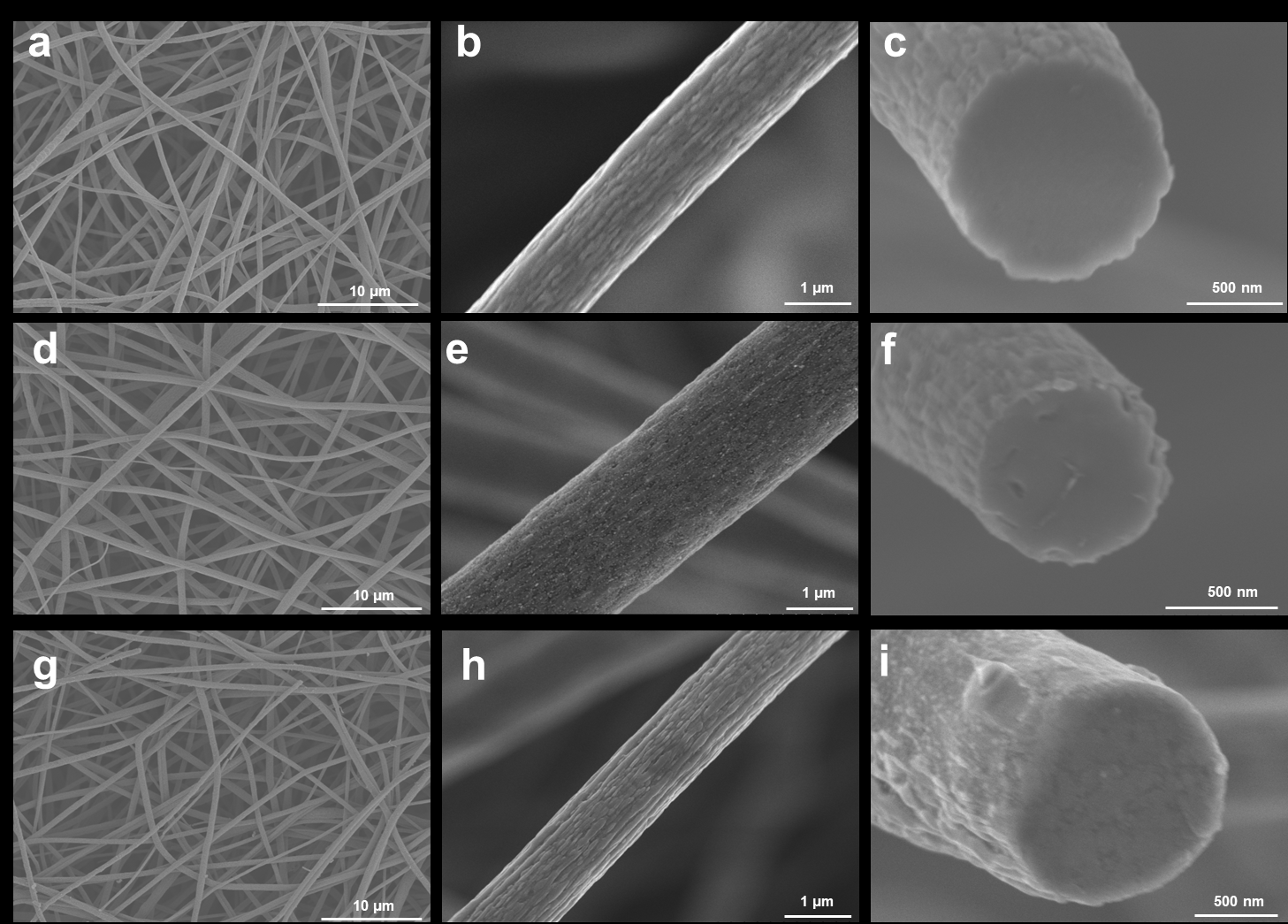


**Fig. S12** The SEM images of Si-Ti-C-O fibrous membranes: (**a-c**) Si-Ti-C-O-1, (**d-f**) Si-Ti-C-O-2, (**g-i**) Si-Ti-C-O-3


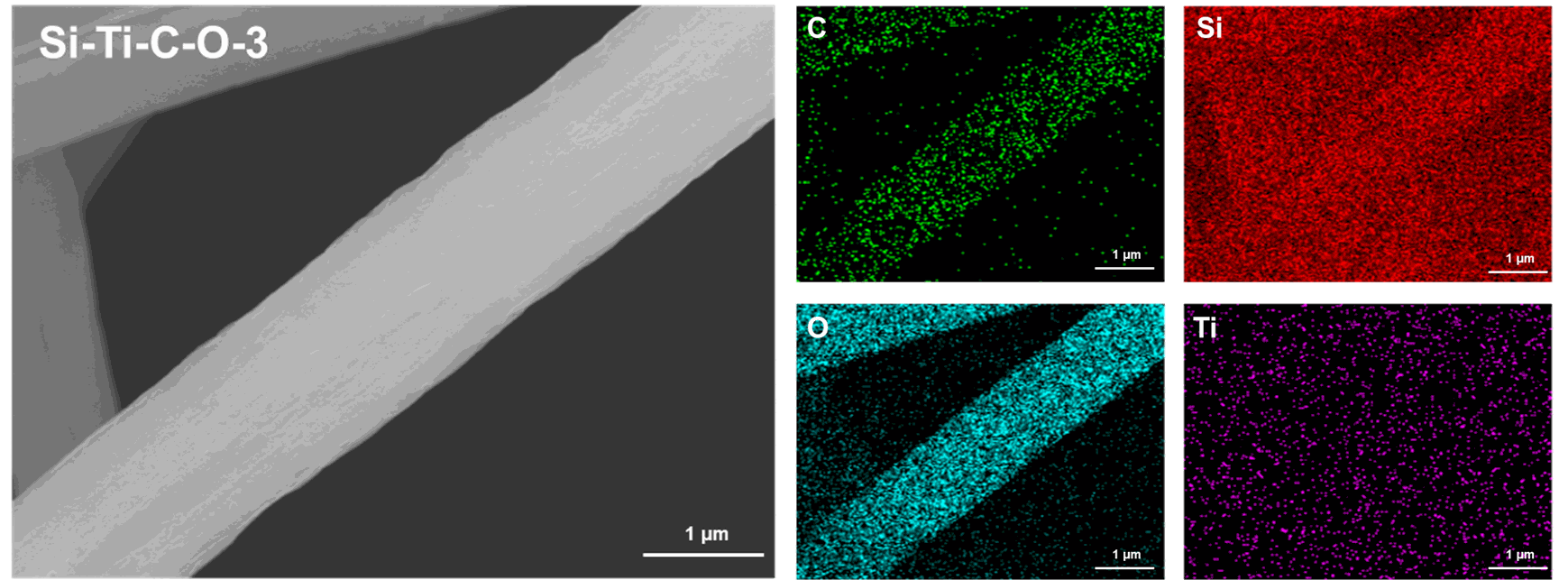


**Fig. S13** Elemental mappings of the Si-Ti-C-O-3 fiber

**Table S2** The elemental content of Si-Ti-C-O-3 fibrous membrane obtained by SEM-EDS

| Element | Si | C | O | Ti |
| --- | --- | --- | --- | --- |
| Content (wt%) | 39.85 | 20.74 | 36.96 | 1.99 |


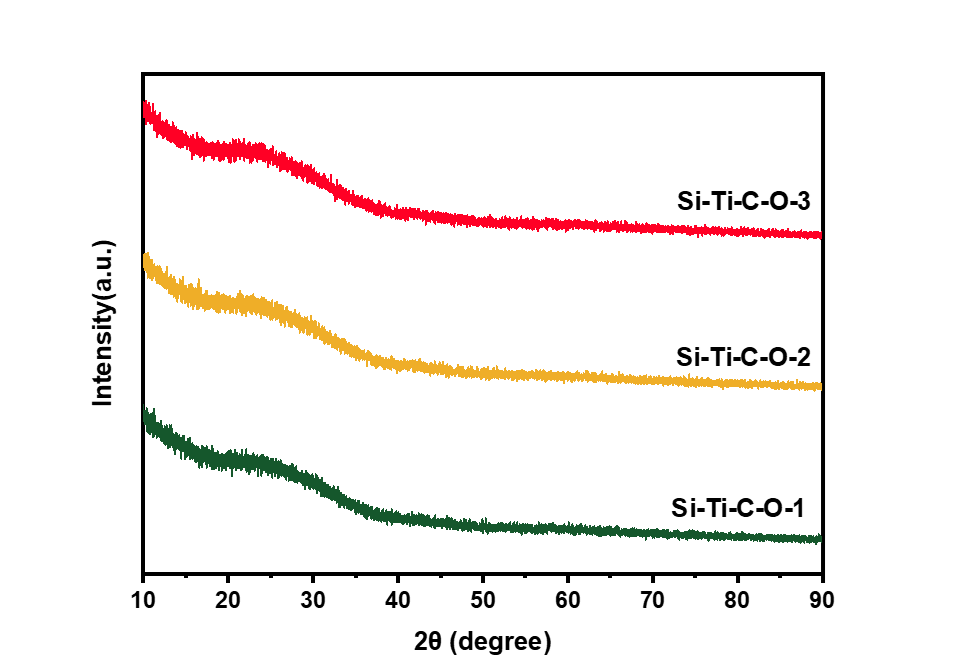


**Fig. S14** XRD curves of Si-Ti-C-O fibrous membranes


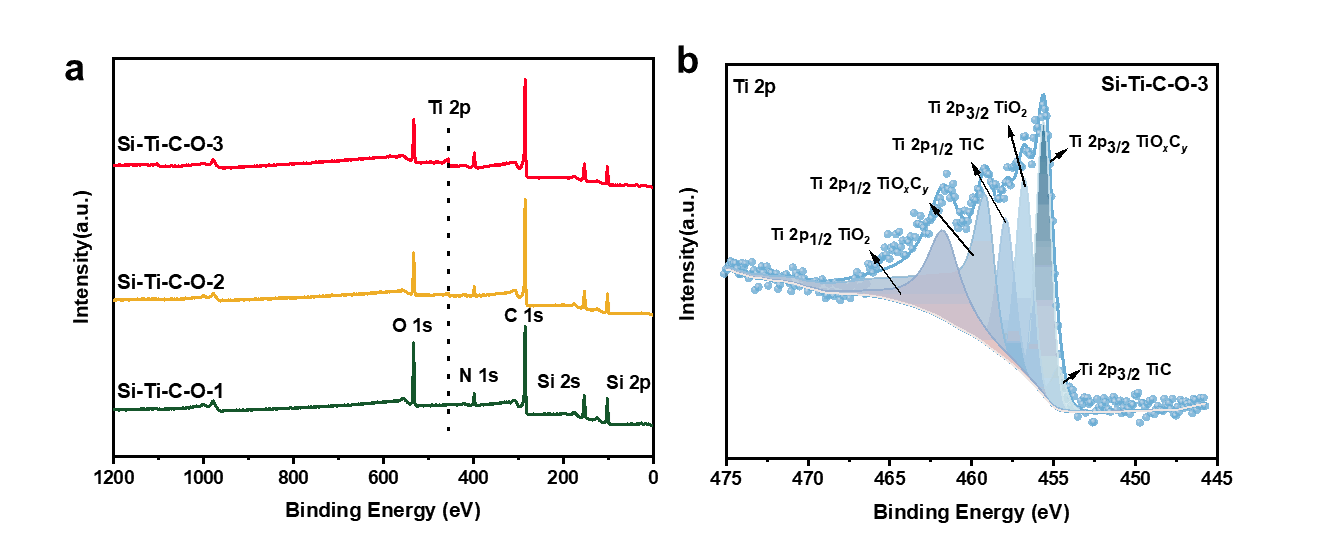


**Fig. S15** (**a**) XPS spectra of Si-Ti-C-O fibrous membranes, and (**b**) Ti 2p spectra of Si-Ti-C-O-3 fibrous membranes


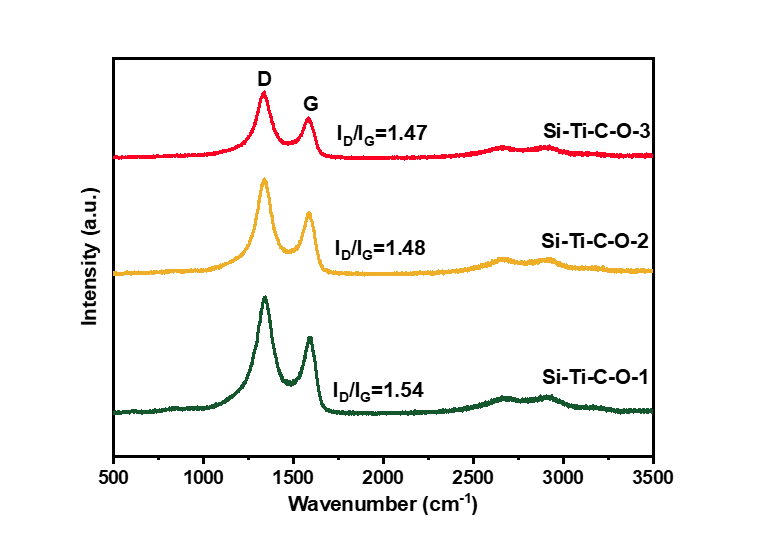


**Fig. S16** Raman spectra of Si-Ti-C-O fibrous membranes

**Table S3** The elemental composition of Si-Ti-C-O fibrous membranes

| Membrane | Si (wt%) | Ti (wt%) | C (wt%) | O (wt%) |
| --- | --- | --- | --- | --- |
| Si-Ti-C-O-1 | 56.17 | 0.47 | 34.98 | 4.26 |
| Si-Ti-C-O-2 | 58.15 | 1.09 | 35.57 | 3.04 |
| Si-Ti-C-O-3 | 52.69 | 2.04 | 30.29 | 2.93 |


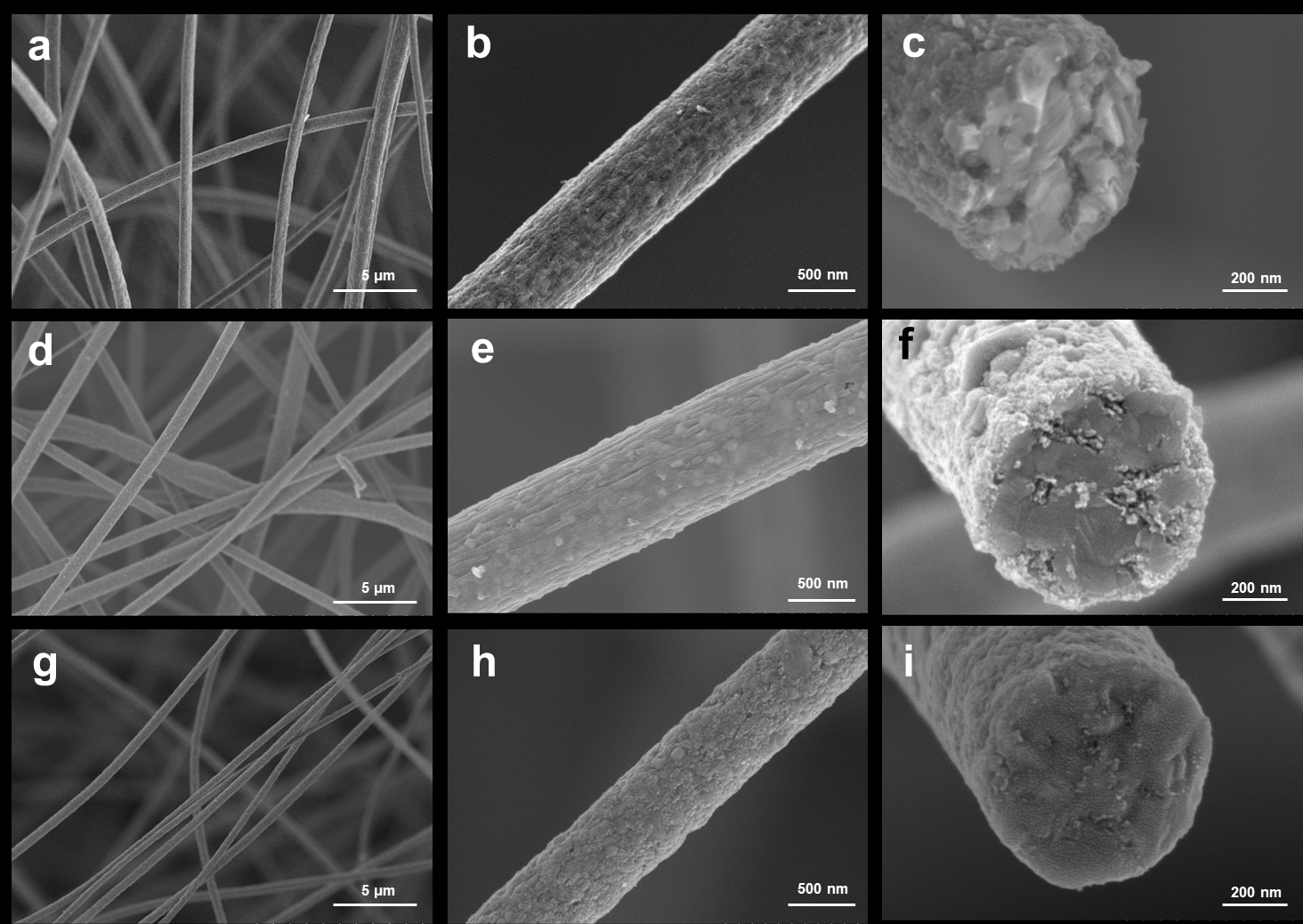


**Fig. S17** SEM images of TiC-SiC fibrous membranes: (**a-c**) TiC-SiC-1, (**d-f**) TiC-SiC-2, (**g-i**) TiC-SiC-3

**Fig. S18** XRD curves of TiC-SiC fibrous membranes

The peaks at 1348 and 1585 cm^-1^ correspond to the D and G peaks of free carbon, respectively. The I_D_/I_G_ radio (the intensity ratio of D peak versus G peak) of Si-Ti-C-O ﬁbers and TiC-SiC ﬁbers are estimated as 1.54 and 0.76, respectively, suggesting that the degree of graphitization of carbon increased after the high-temperature sintering.

**Fig. S19** Raman spectra of TiC-SiC fibrous membranes

**Fig. S20** XPS spectra of TiC-SiC fibrous membranes

**Table S4** The elemental composition of TiC-SiC fibrous membranes

| Membrane | Si (wt%) | Ti (wt%) | C (wt%) | O (wt%) |
| --- | --- | --- | --- | --- |
| TiC-SiC-1 | 48.62 | 0.50 | 39.02 | 0.34 |
| TiC-SiC-2 | 51.80 | 1.06 | 39.86 | 0.32 |
| TiC-SiC-3 | 49.82 | 1.95 | 39.76 | 0.39 |

According to the C 1s spectrum fitting results, the peak at 284.8 eV is attributed to the sp2 bond of free carbon, the peak at 283.5 eV is attributed to the C-Si bond of SiC, the peak at 282.8 eV comes from the C signal of C-Ti, and the peak at 286.0 eV comes from the C signal of C-O, which could be attributed to the residual O element inside the fiber [S1-S4].

**Fig. S21** C 1s spectrum of the TiC-SiC-3 fibrous membrane





**Fig. S22** SAED pattern of the TiC-SiC fiber


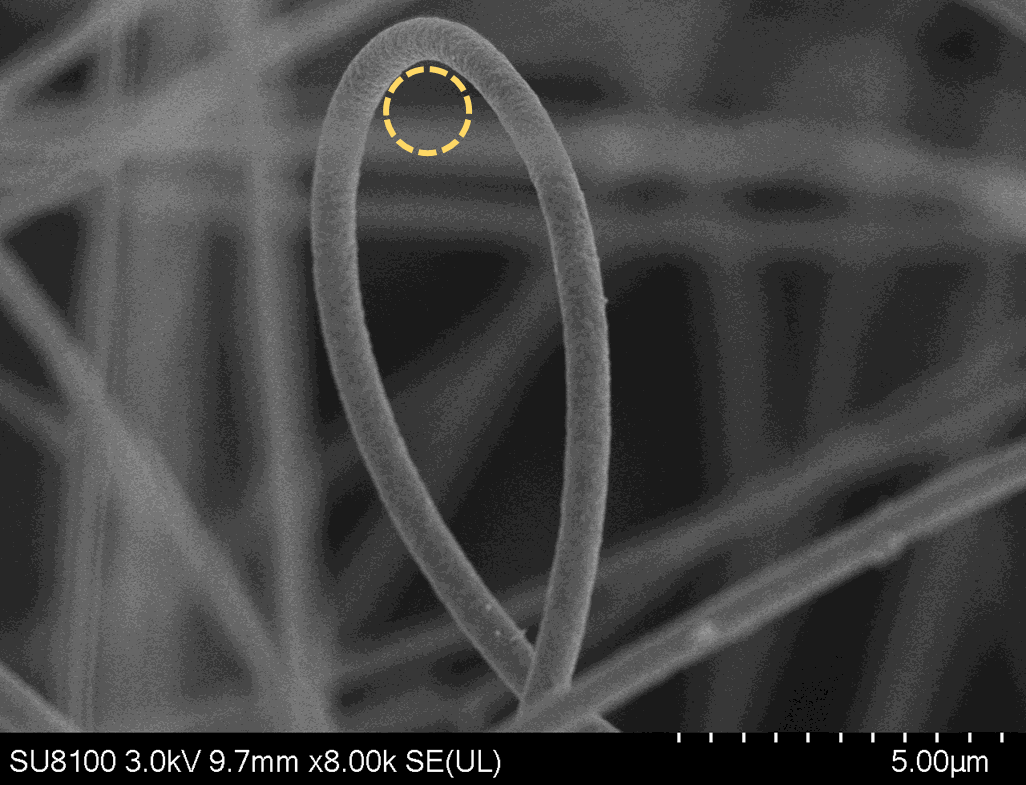


**Fig. S23** SEM image of the bended single TiC-SiC fiber


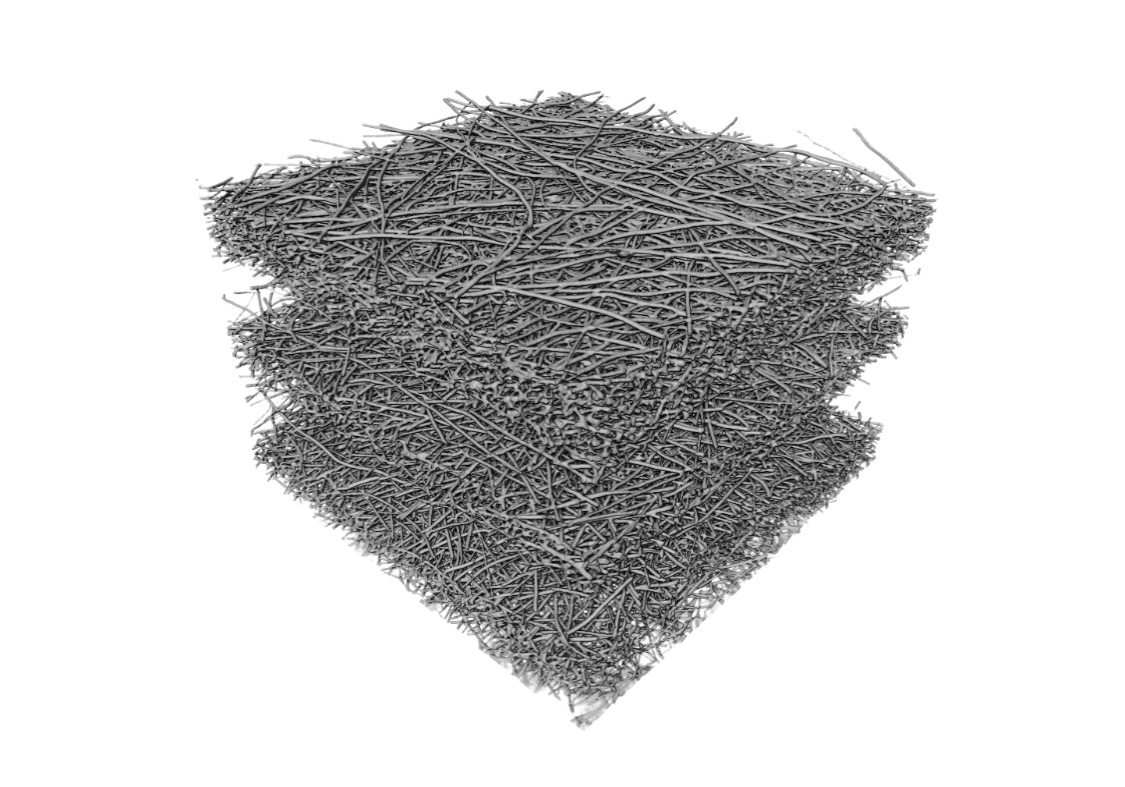


**Fig. S24** The three-dimensional CT image reconstruction of TiC-SiC fibrous membrane


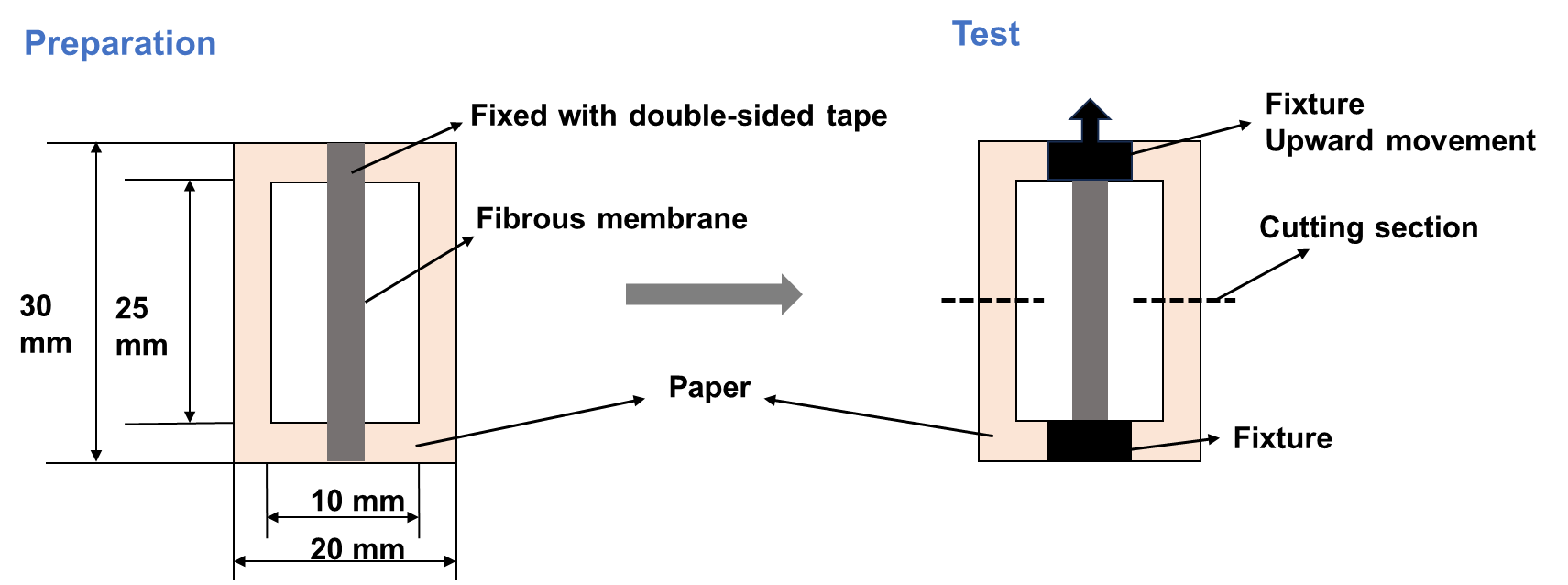


**Fig. S25** Schematic of the mechanical property test for the fibrous membranes


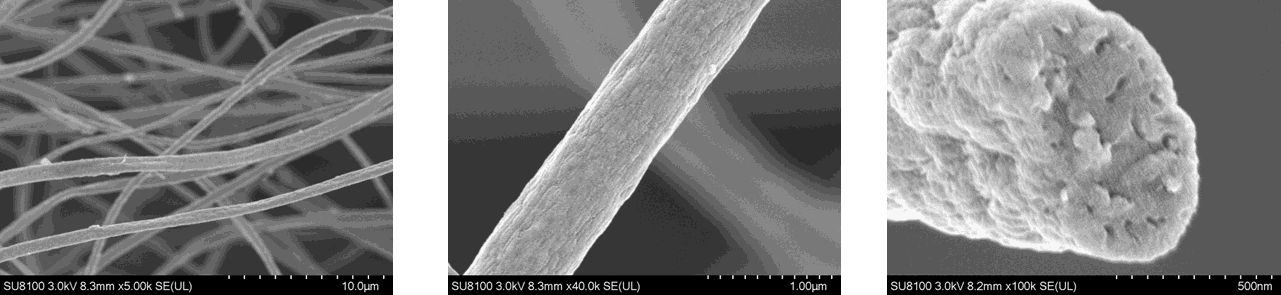


**Fig. S26** SEM image of SiC fibrous membrane

**Fig. S27** XRD pattern of SiC fibrous membrane


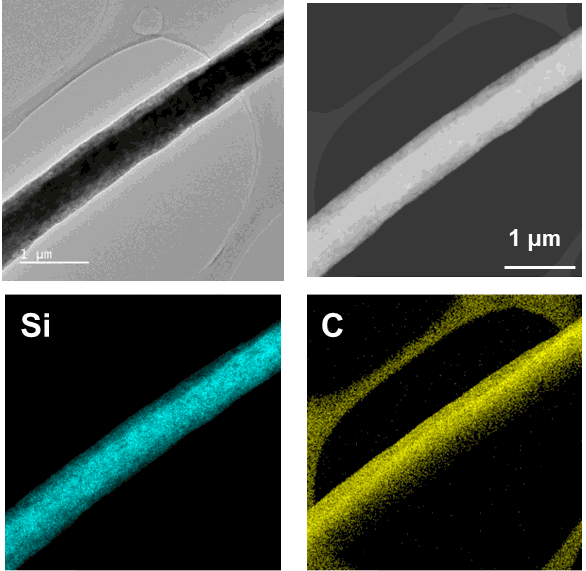


**Fig. S28** TEM and elemental mapping images of the SiC fiber without Ti

**Fig. S29** The average strength of different fibrous membranes

**Fig. S30** The stress-strain curves of the mechanical tests for 5 times for the TiC-SiC-3 fibrous membrane


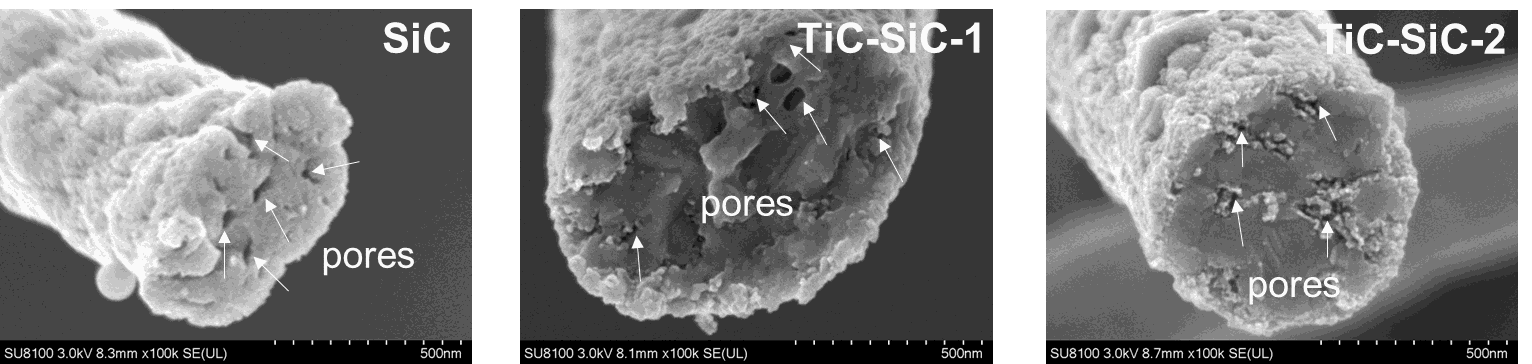


**Fig. S31** SEM images of the cross section of the fibers in different membrane

**Table S5** The specific surface area and pore volume of the fibers

| Sample | BET Surface Area (m^2^/g) | Single point adsorption total pore volume of pores (cm^3^/g) | BJH Adsorption cumulative volume of pores (cm^3^/g) | BJH Desorption cumulative volume of pores (cm^3^/g) |
| --- | --- | --- | --- | --- |
| SiC | 16.7611 | 0.039617 | 0.035449 | 0.038515 |
| TiC-SiC-1 | 22.3529 | 0.048533 | 0.047291 | 0.048505 |
| TiC-SiC-2 | 36.8877 | 0.194705 | 0.193978 | 0.193952 |
| TiC-SiC-3 | 3.9719 | 0.024959 | 0.023256 | 0.024967 |


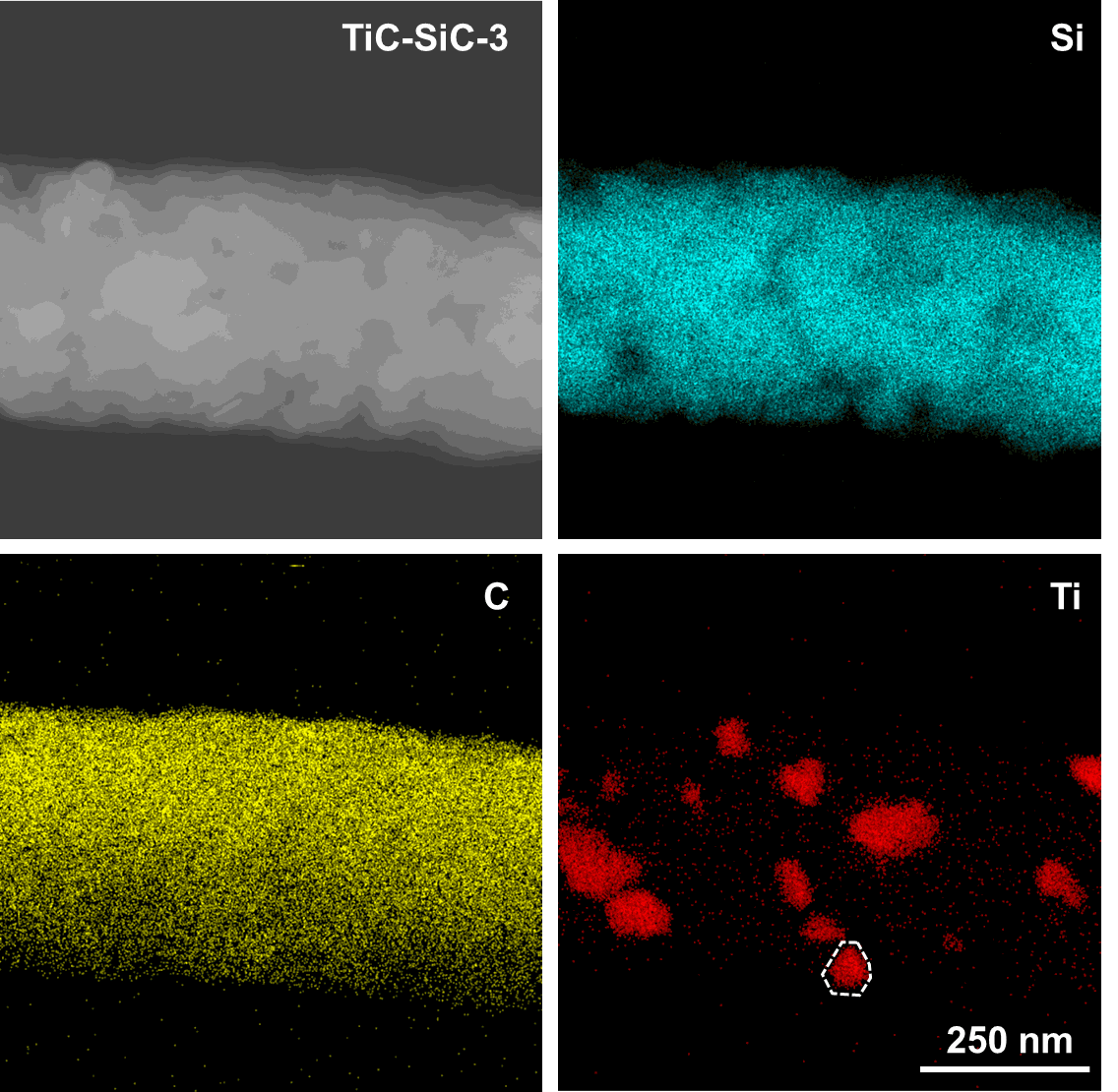


**Fig. S32** Elemental mapping images of TiC-SiC-3 fiber


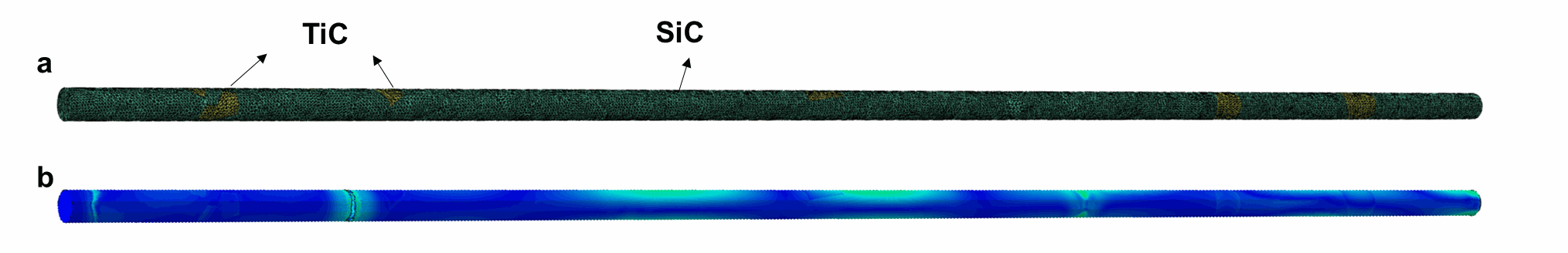


**Fig. S33** (**a**) Model diagram of finite element analysis, (**b**) TiC-SiC fiber fracture stress distribution map


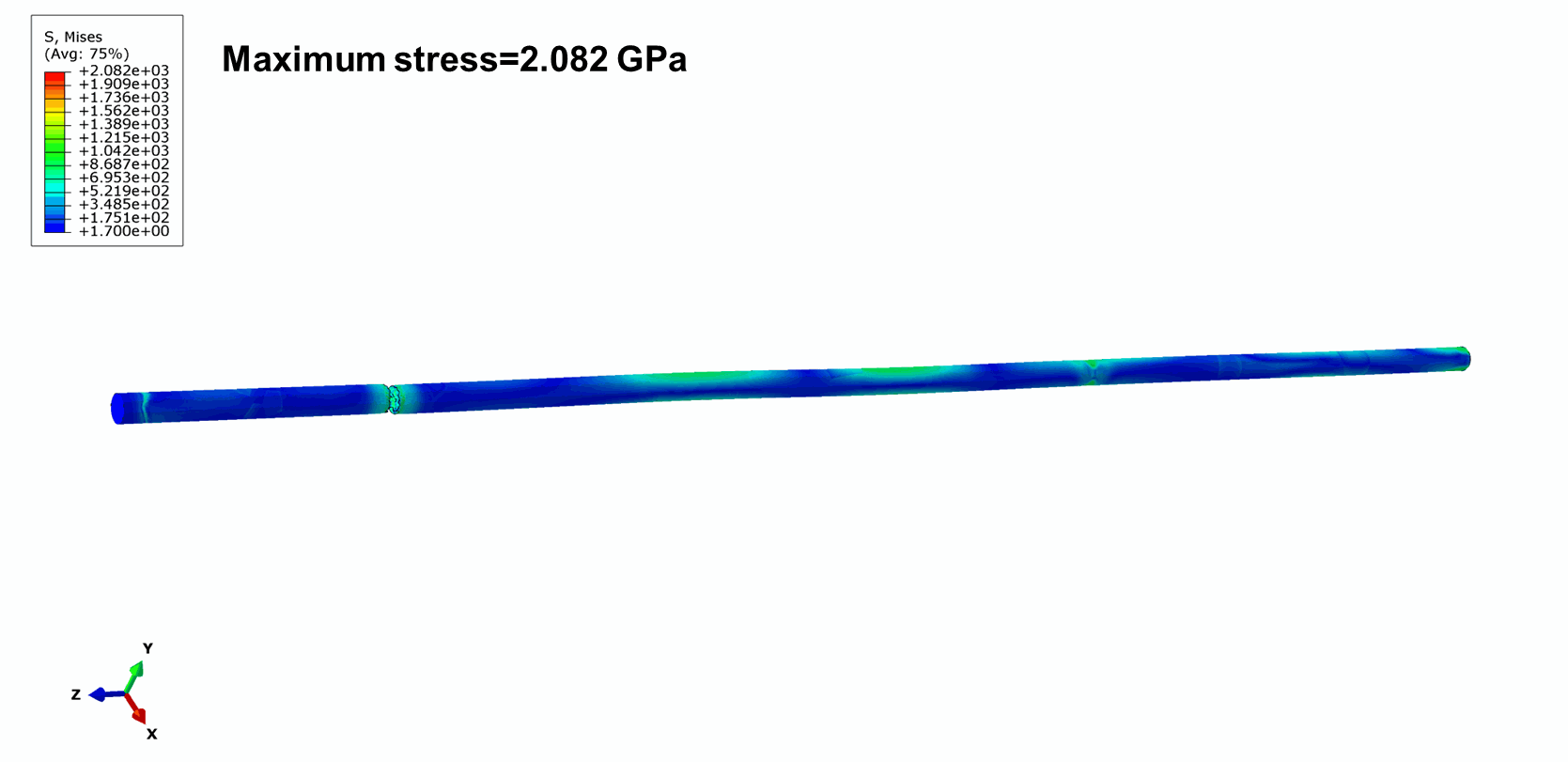


**Fig. S34** Stress distribution of TiC-SiC fiber


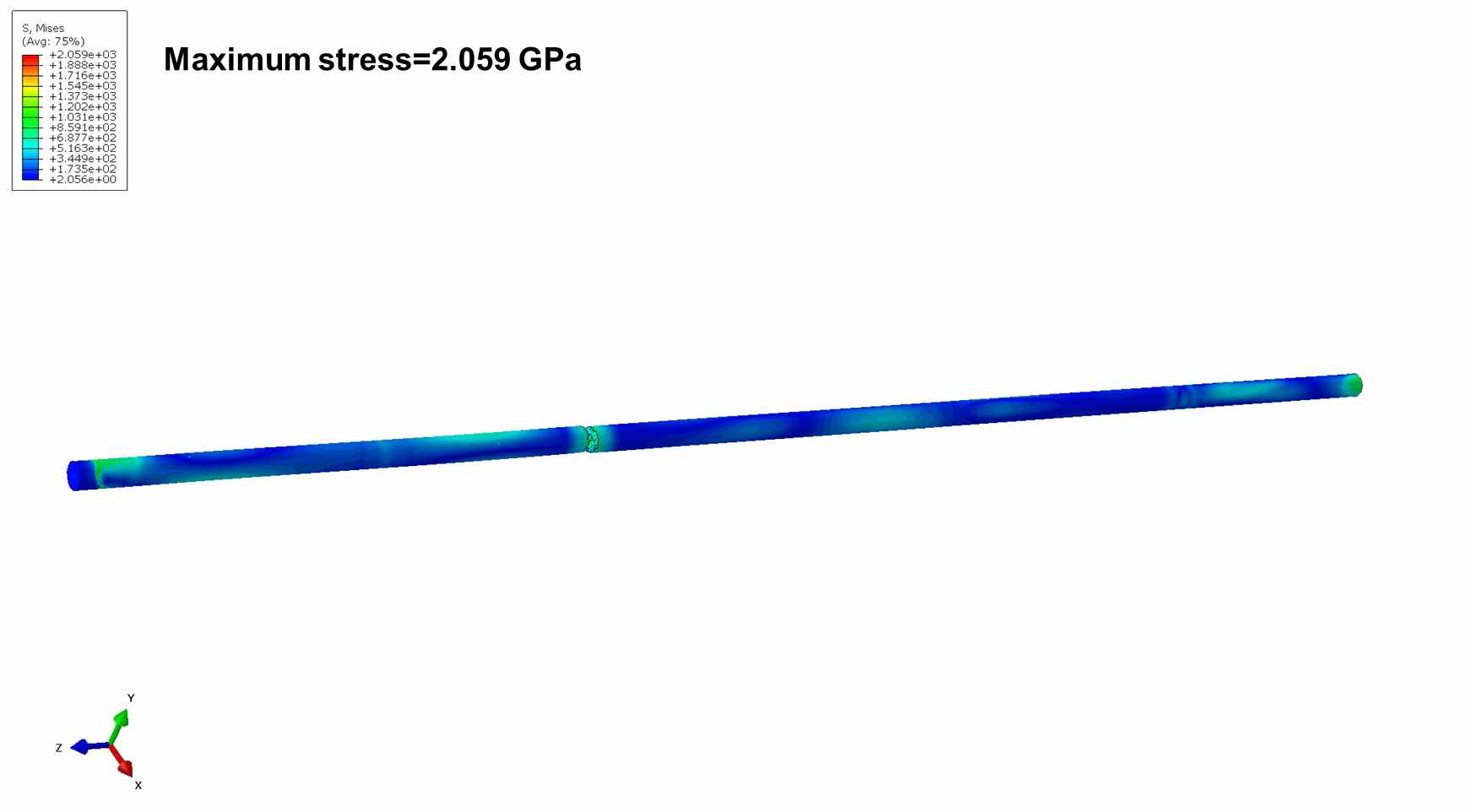


**Fig. S35** Stress distribution of SiC fiber

**Fig. S36** Fracture curves from molecular dynamics simulations


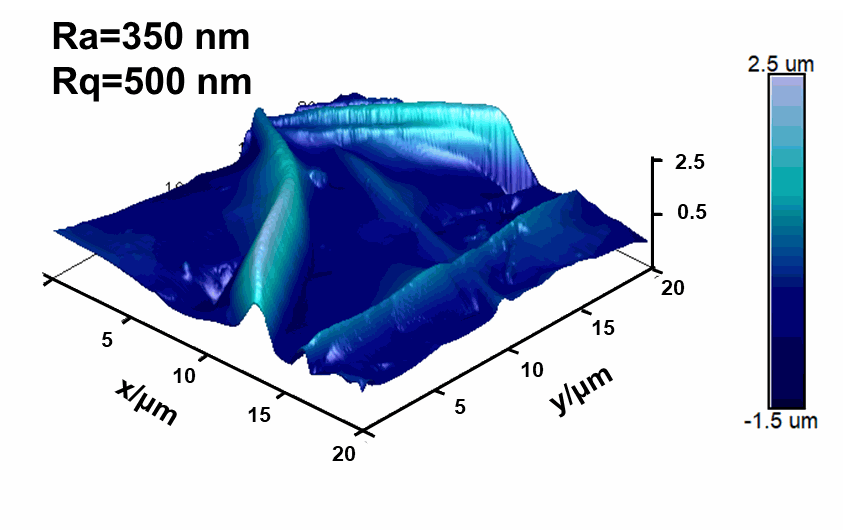


**Fig. S37** AFM image of the surface of the SiC fibrous membrane


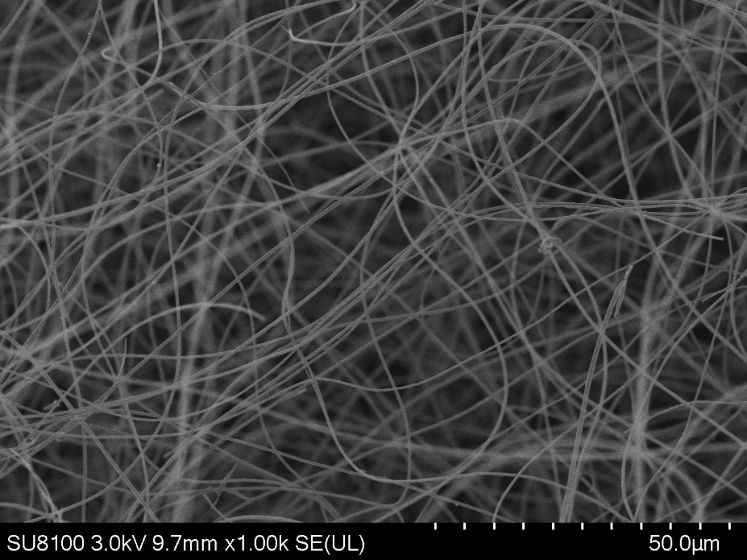


**Fig. S38** SEM image of the TiC-SiC ultrafine fibers in the membrane


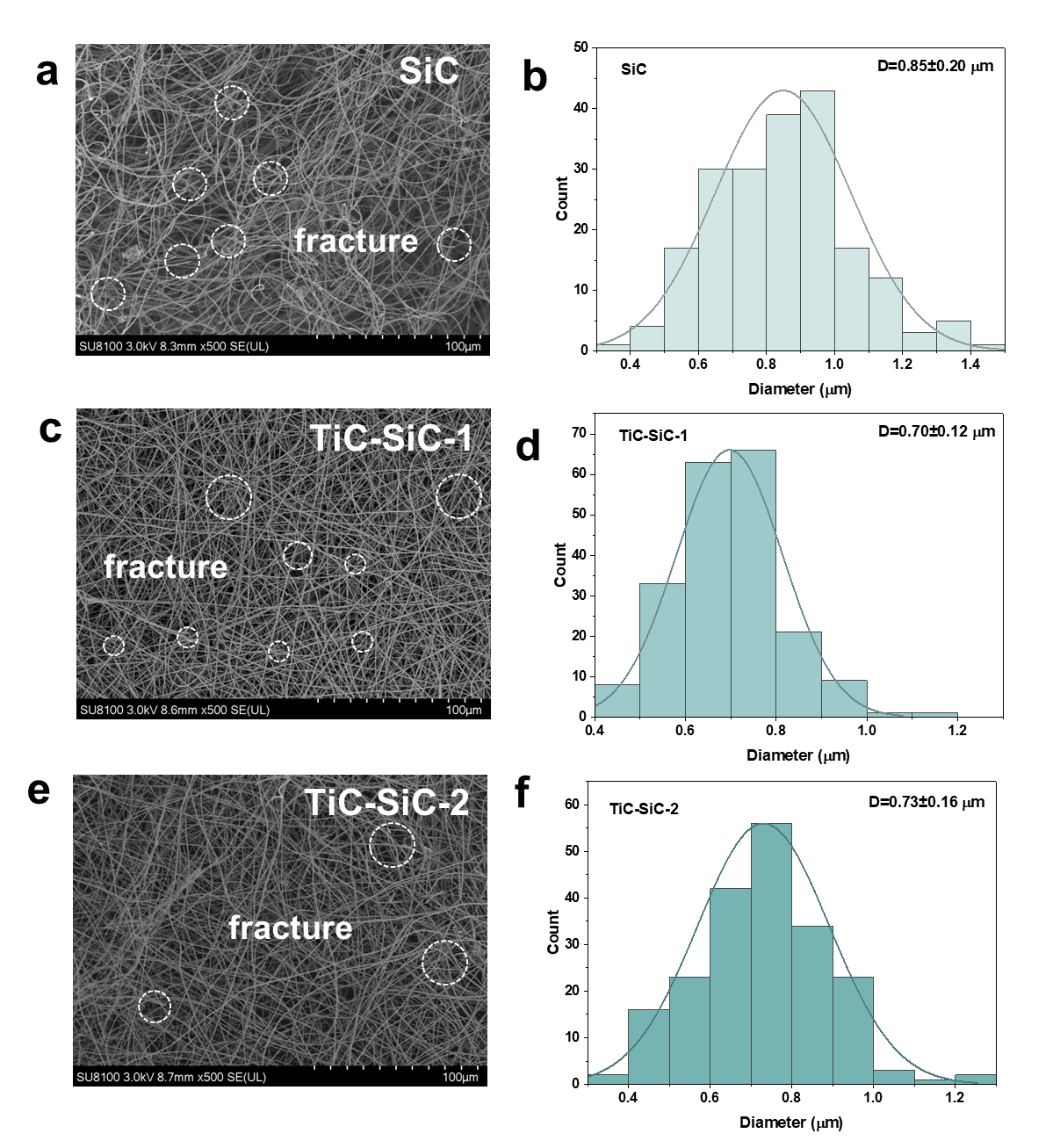


**Fig. S****39** SEM images and the statistics of diameter distribution: (**a-b**) SiC fibrous membrane, (**c-d**) TiC-SiC-1 fibrous membrane, (**e-f**) TiC-SiC-2 fibrous membrane


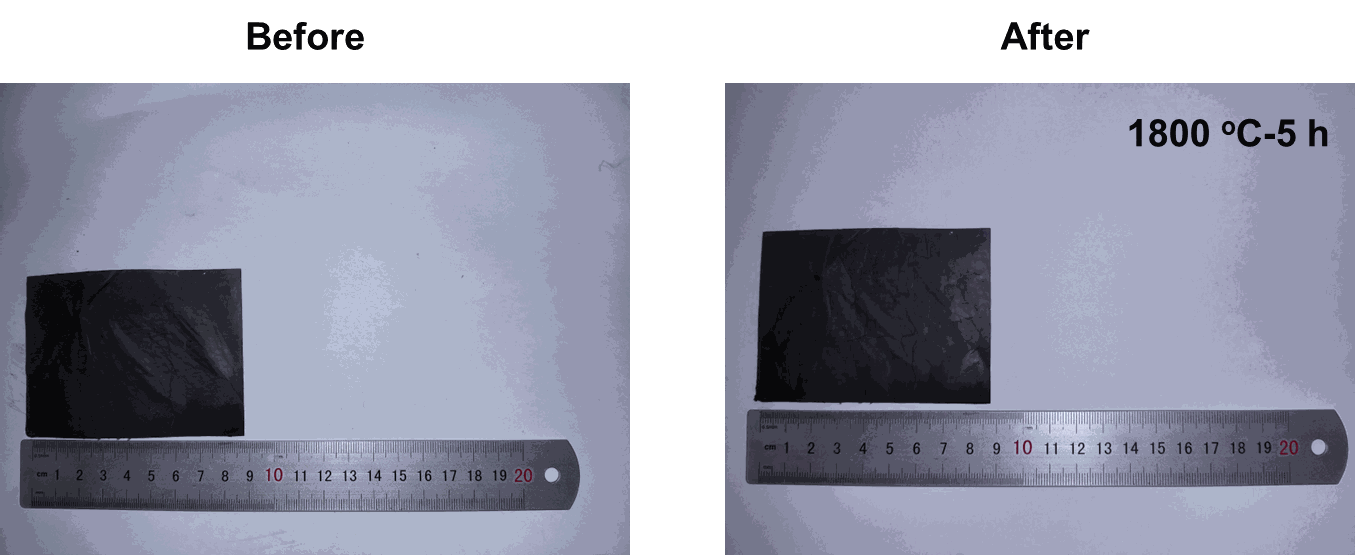


**Fig. S40** Optical photograph of TiC-SiC fibrous membrane before and after being heated at 1800 ℃ for 5 h in argon atmosphere


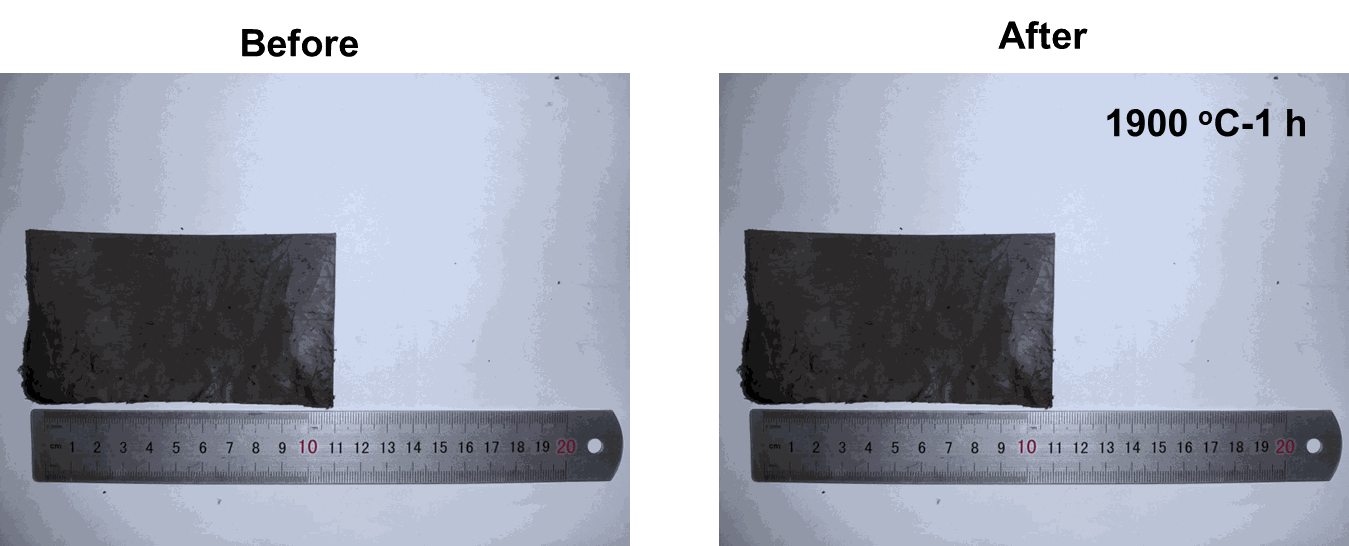


**Fig. S41** Optical photograph of TiC-SiC fibrous membrane before and after being heated at 1900 ℃ for 1 h in argon atmosphere


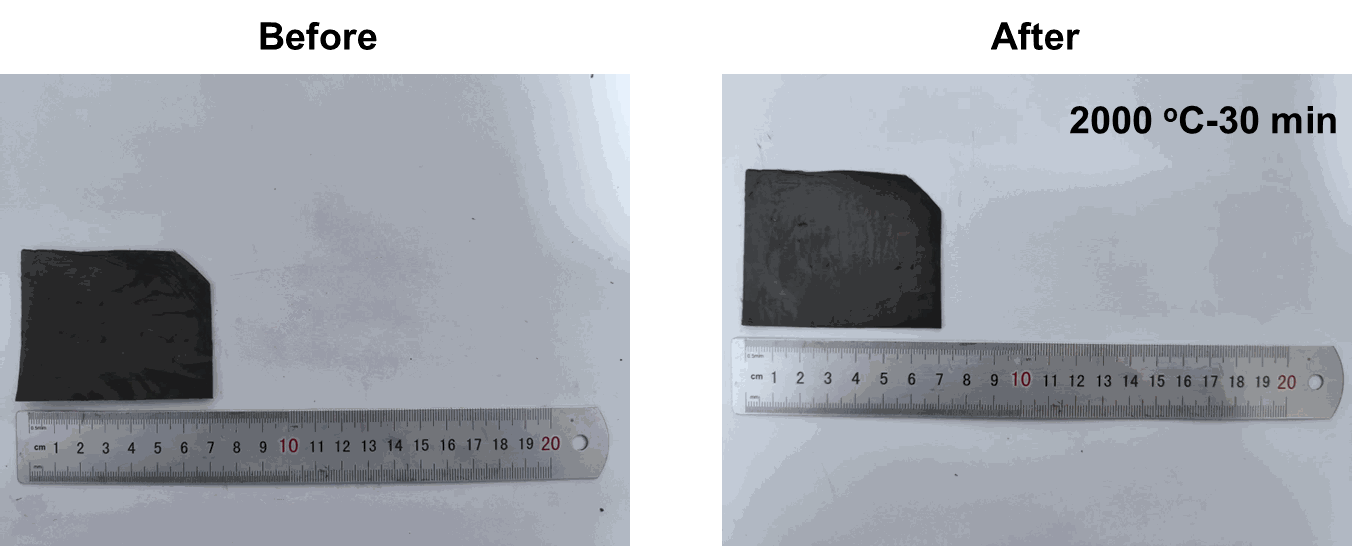


**Fig. S42** Optical photograph of TiC-SiC fibrous membrane before and after being heated at 2000 ℃ for 30 min in argon atmosphere


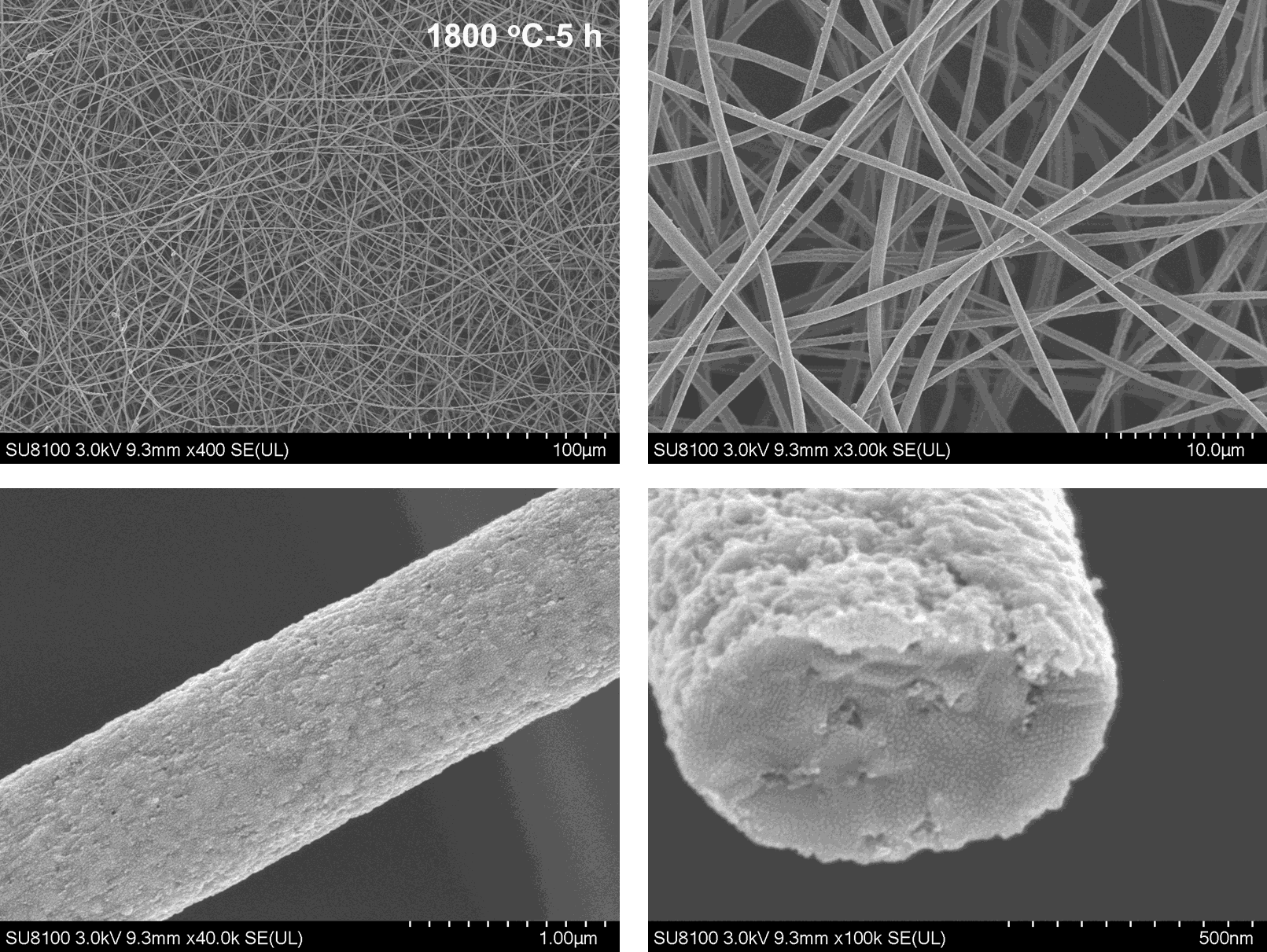


**Fig. S43** SEM images of TiC-SiC fibrous membrane after heat treatment at 1800 ℃ for 5 h in argon atmosphere


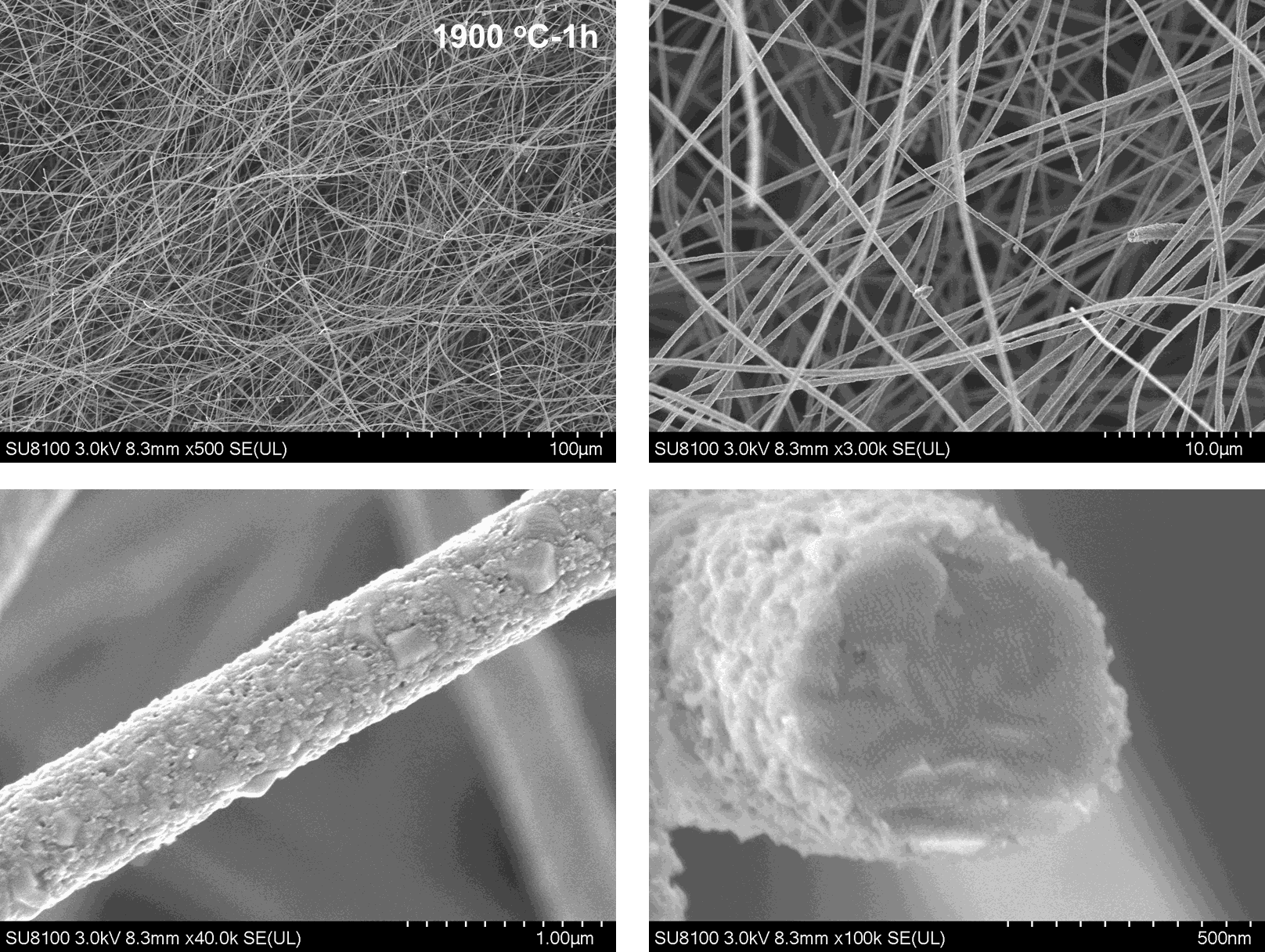


**Fig. S44** SEM images of TiC-SiC fibrous membrane after heat treatment at 1900 ℃ for 1 h in argon atmosphere


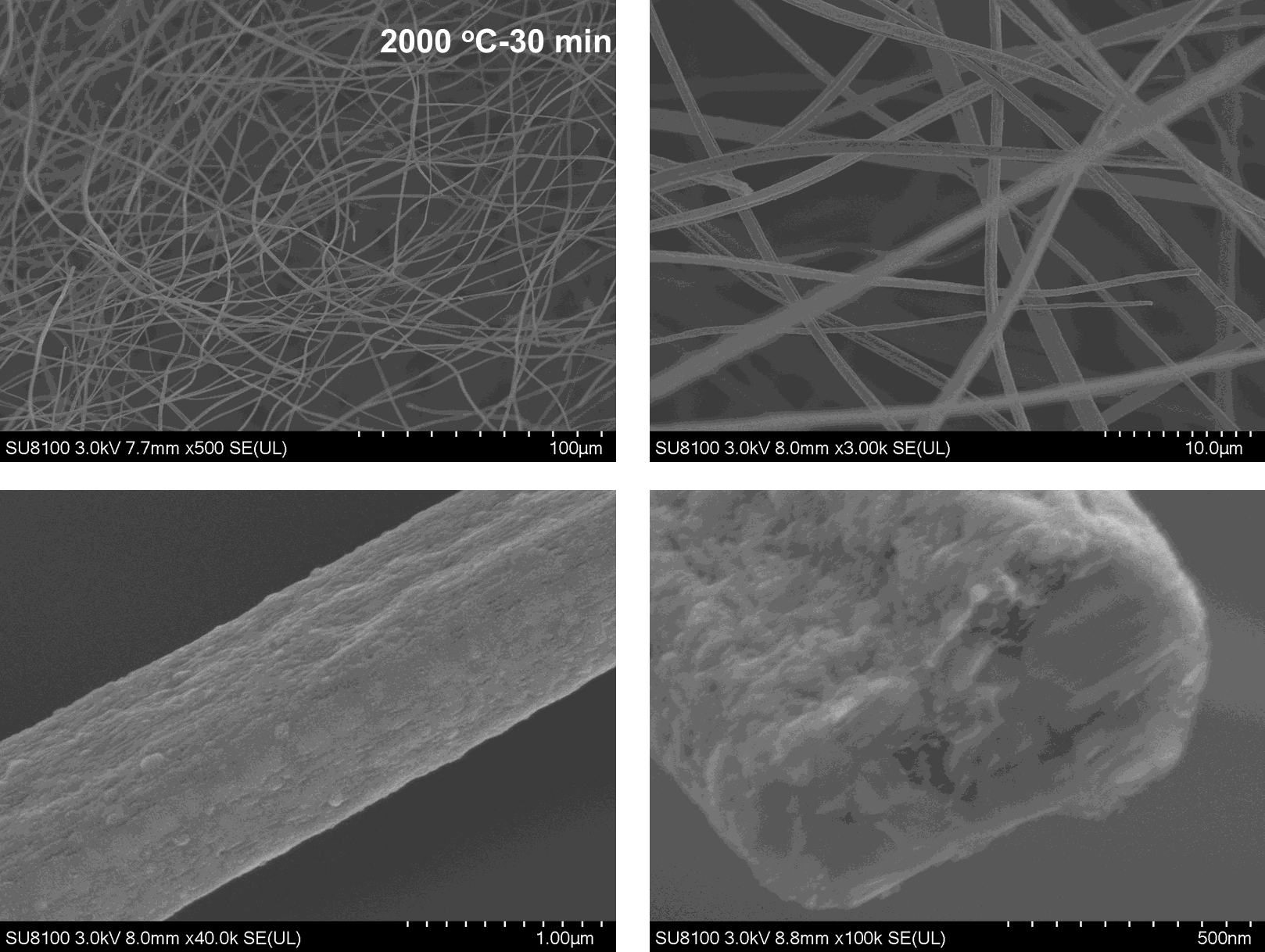


**Fig. S45** SEM images of TiC-SiC fibrous membrane after heat treatment at 2000 ℃ for 30 min


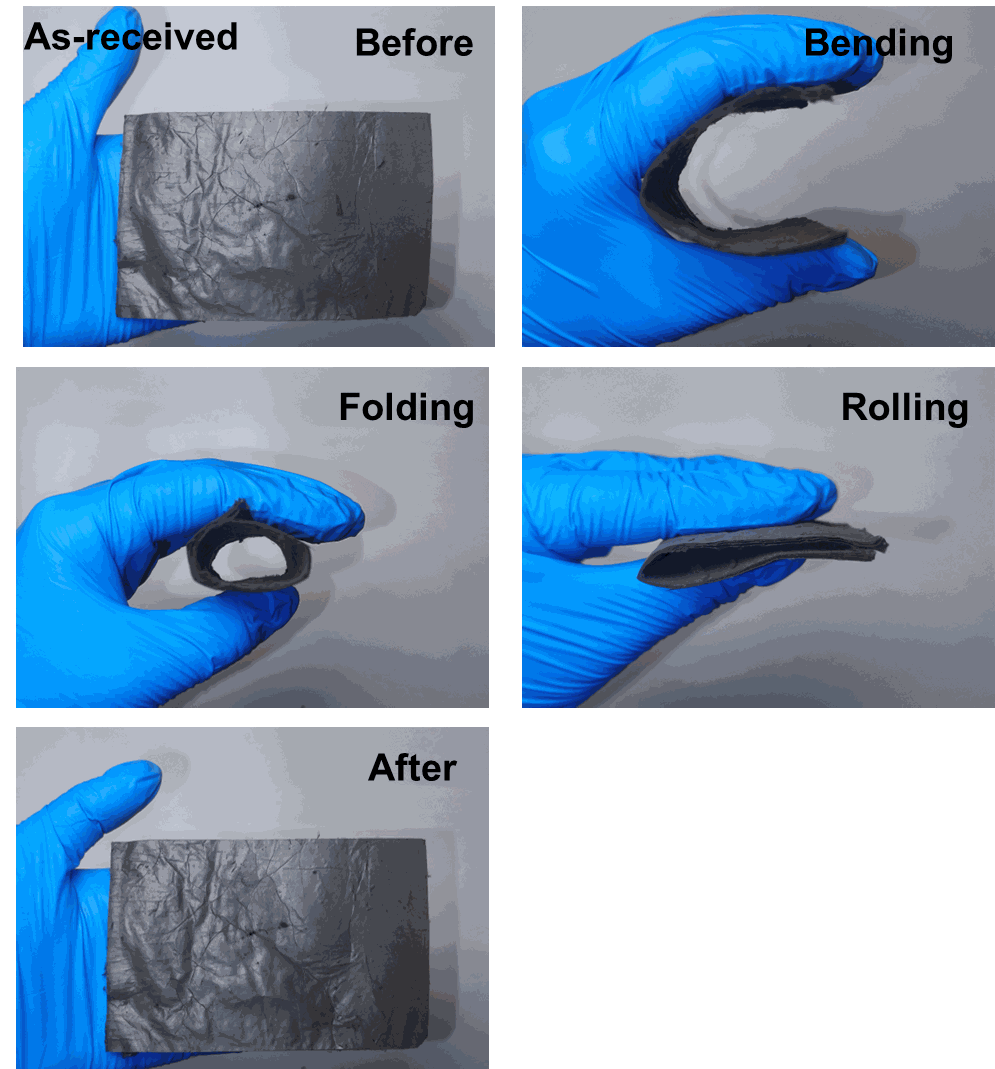


**Fig. S46** Demonstration of the flexibility of the original TiC-SiC fibrous membrane


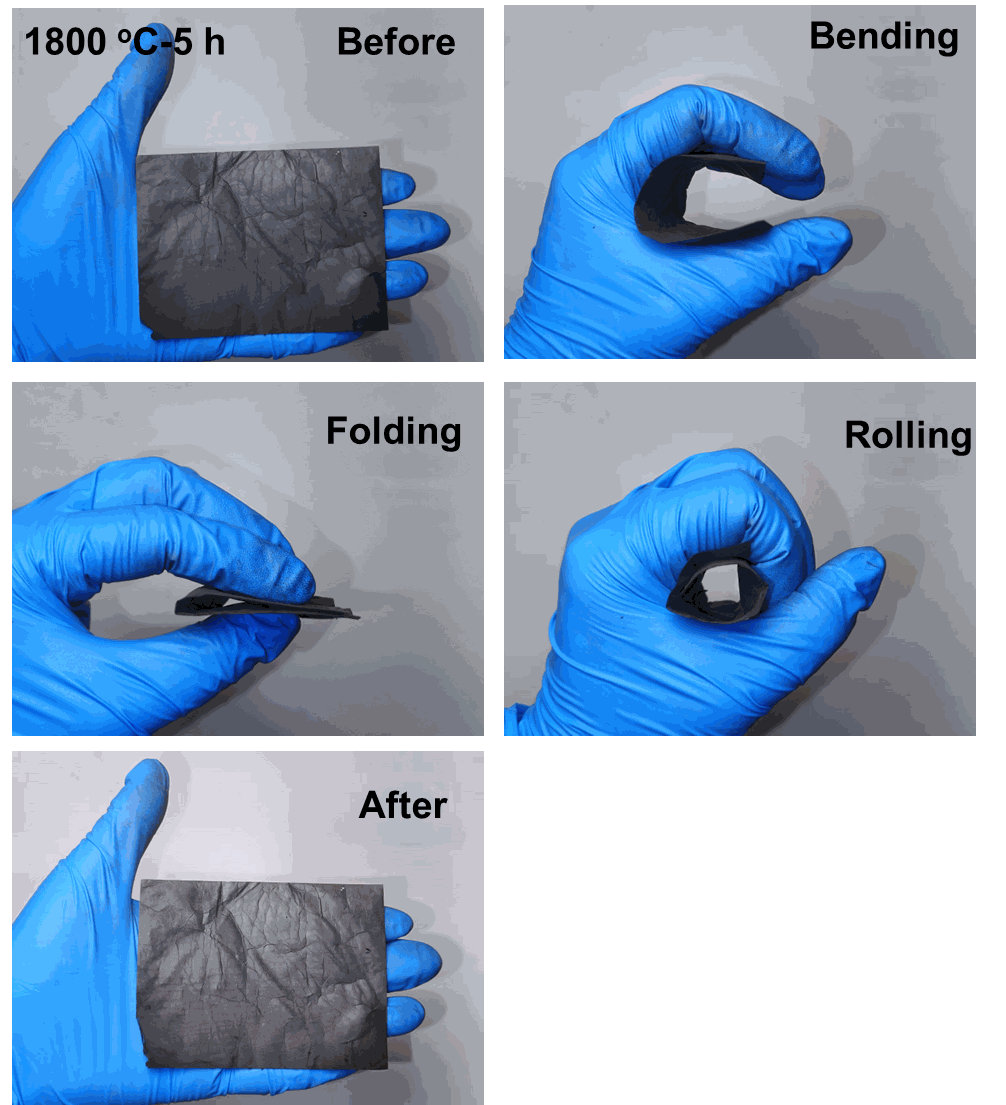


**Fig. S47** Demonstration of the flexibility of the TiC-SiC fibrous membrane after heat treatment at 1800 ℃ for 5 h in argon atmosphere


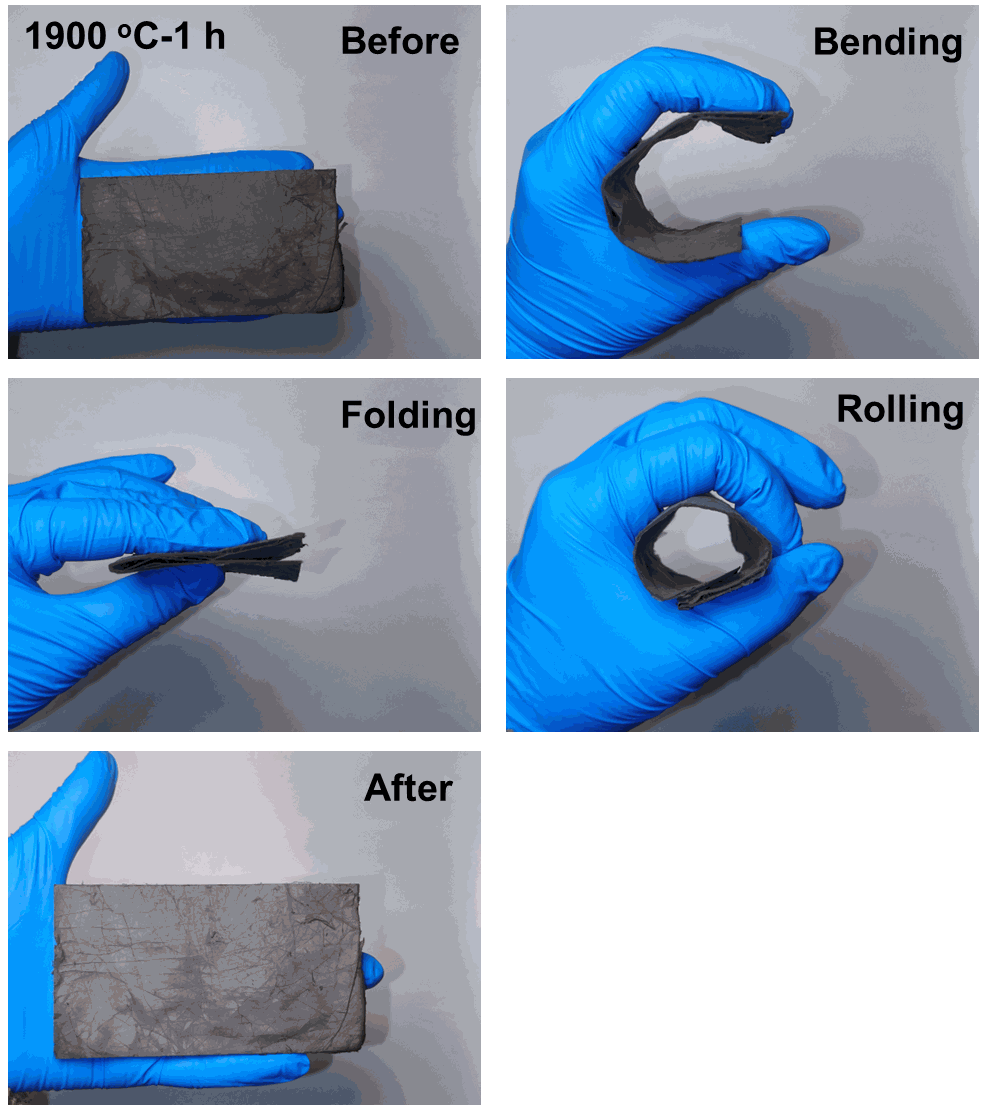


**Fig. S48** Demonstration of the flexibility of the TiC-SiC fibrous membrane after heat treatment at 1900 ℃ for 1 h in argon atmosphere


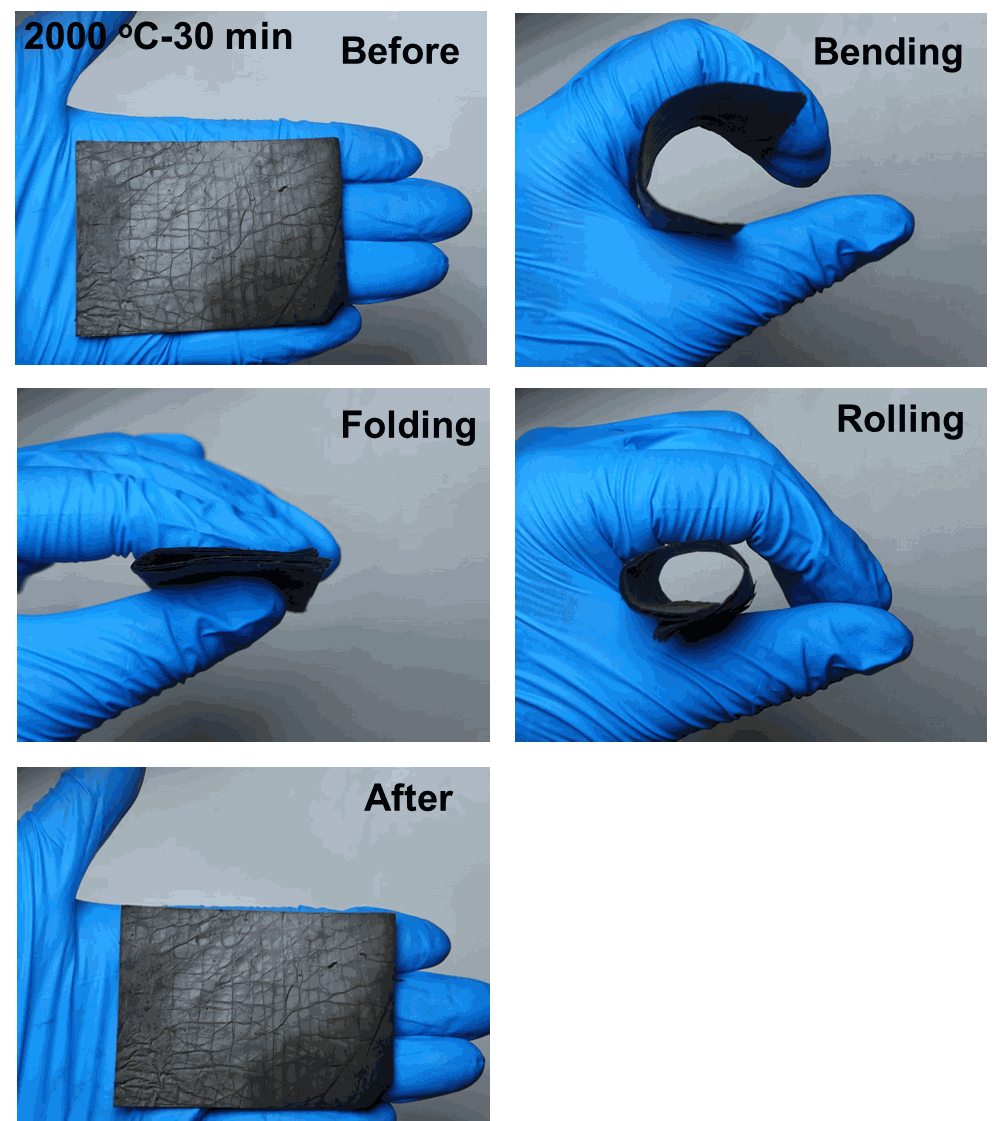


**Fig. S49** Demonstration of the flexibility of the TiC-SiC fibrous membrane after heat treatment at 2000 ℃ for 30 min in argon atmosphere

**Table S6** Comparison of maximum working temperature and tensile strength of TiC-SiC fibrous membranes and different membranes previously reported in literature

| Materials | Max working Temperature (^o^C) | Tensile strength (MPa) | Refs. |
| --- | --- | --- | --- |
| montmorillonite@ZrO_2_-SiO_2_ membranes | ~1000 | 1.83 | [S5] |
| TiO_2_ nanofibrous membranes | 800 | 0.62 (Max) | [S6] |
| CNF-CNNS | 1700 | ~1.9 | [S7] |
| OUHS 3YSZ fibrous membranes | 1500 | ~3.5 (average) | [S8] |
| OUHS TiO_2_ fibrous membranes | 1300 | ~1.2 | [S8] |
| OUHS HfO_2_ fibrous membranes | 1500 | ~1.6 | [S8] |
| OUHS Al_2_O_3_ fibrous membranes | 1300 | ~2.0 | [S8] |
| OUHS Y_2_O_3_ fibrous membranes | 1400 | ~1.2 | [S8] |
| MgAl_2_O_4_ nanofibrous membranes | 1600 | 1.94 | [S9] |
| SiZrOC nanofibrous membranes | 1200 | 0.82 (Max) | [S10] |
| Ultrafine SiC fiber | 1900 | ~0.32 (Max) | [S11] |
| This work | 2000 | 2.1 |  |

It was noted that:

1.CNF-CNNS: 2D ceramic nanofibrous membranes with a crosslinked nanofiber network structure (Al_2_O_3_-SiO_2_ nanofiber membrane)

2.OUHS: open ultrafast high-temperature sintering (heating/cooling rate = 10000 K min^−1^)

3YSZ: 3 mol% yttria-stabilized zirconia

3.The maximum operating temperature of the OUHZ 3YSZ fiber membranes fibers were not explicitly provided, therefore the preparation temperature at its highest strength is selected.


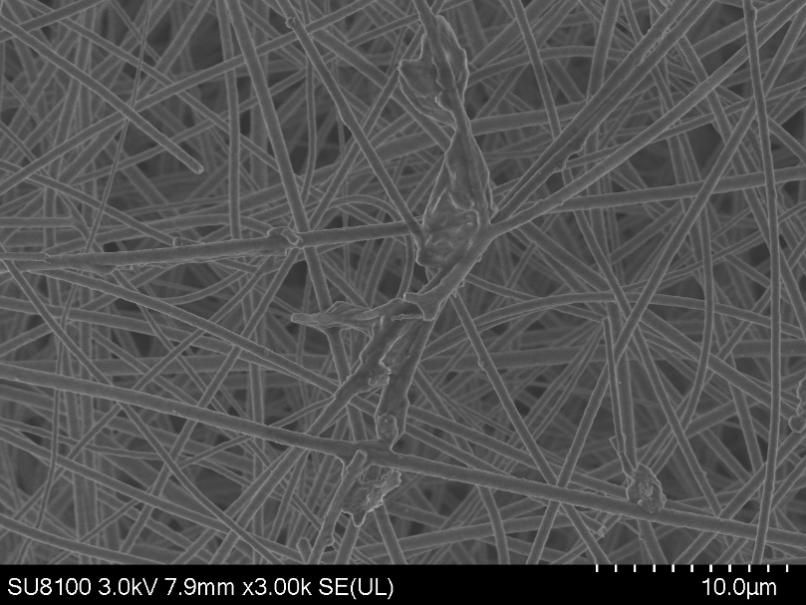


**Fig. S50** SEM images of the TiC-SiC fibrous membrane after heat treatment at 1200 ℃ for 1 h in air atmosphere


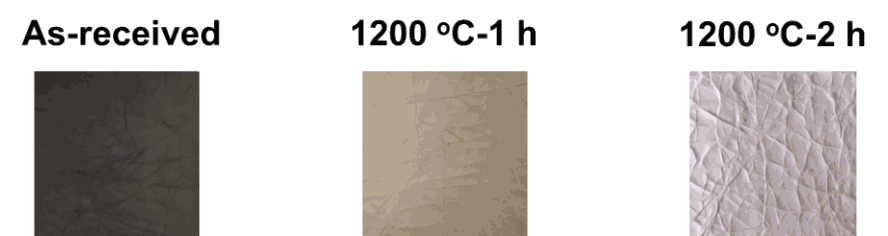


**Fig. S51** Optical photos of the TiC-SiC fibrous membrane before and after oxidation at 1200 ^o^C in air

**Fig. S52** The stress–strain curve of the TiC-SiC ultrafine fibrous membrane after oxidation at 1200 ℃ for 2 h

**Fig. S53** XRD pattern of the TiC-SiC fibrous membrane treated at 1200 ℃ for 2 h in air


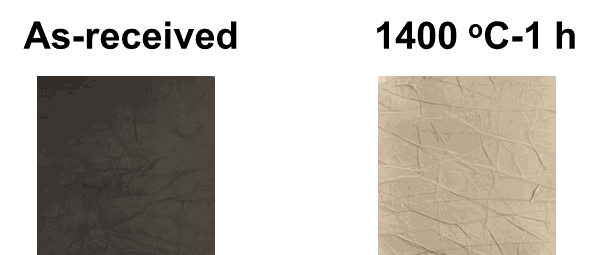


**Fig. S54** Optical photos of the TiC-SiC fibrous membrane before and after oxidation at 1400 ℃ for 1 h in air

**Fig. S55** XRD pattern of the TiC-SiC fibrous membrane treated at 1400 ℃ for 1 h in air

**Fig. S56** The stress–strain curve of the TiC-SiC fibrous membrane after oxidation at 1400 ℃ for 1 h


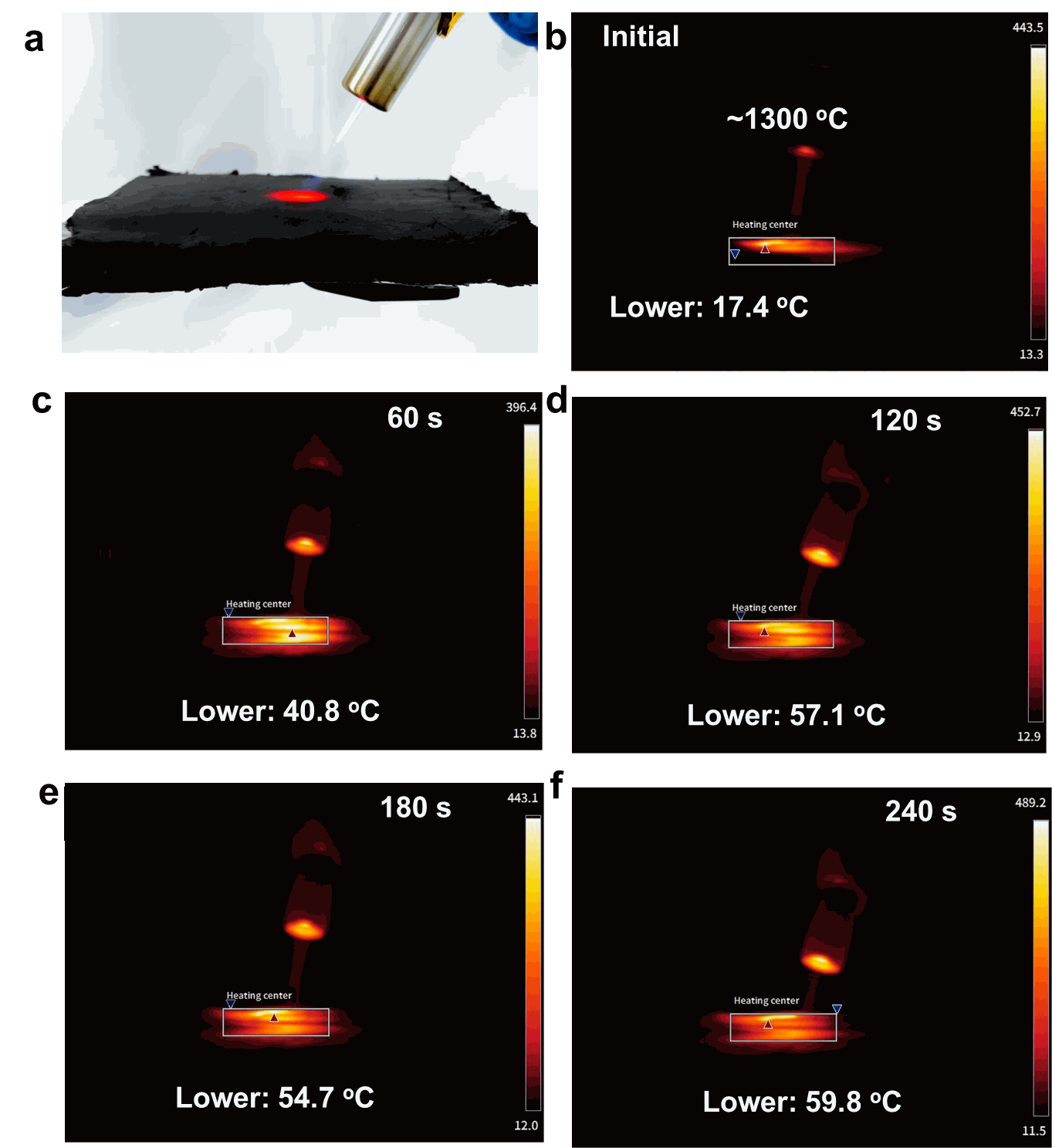


**Fig. S57** Optical photos of (**a**) the device heated by butane flame and (**b-f**) infrared photos during the heating process


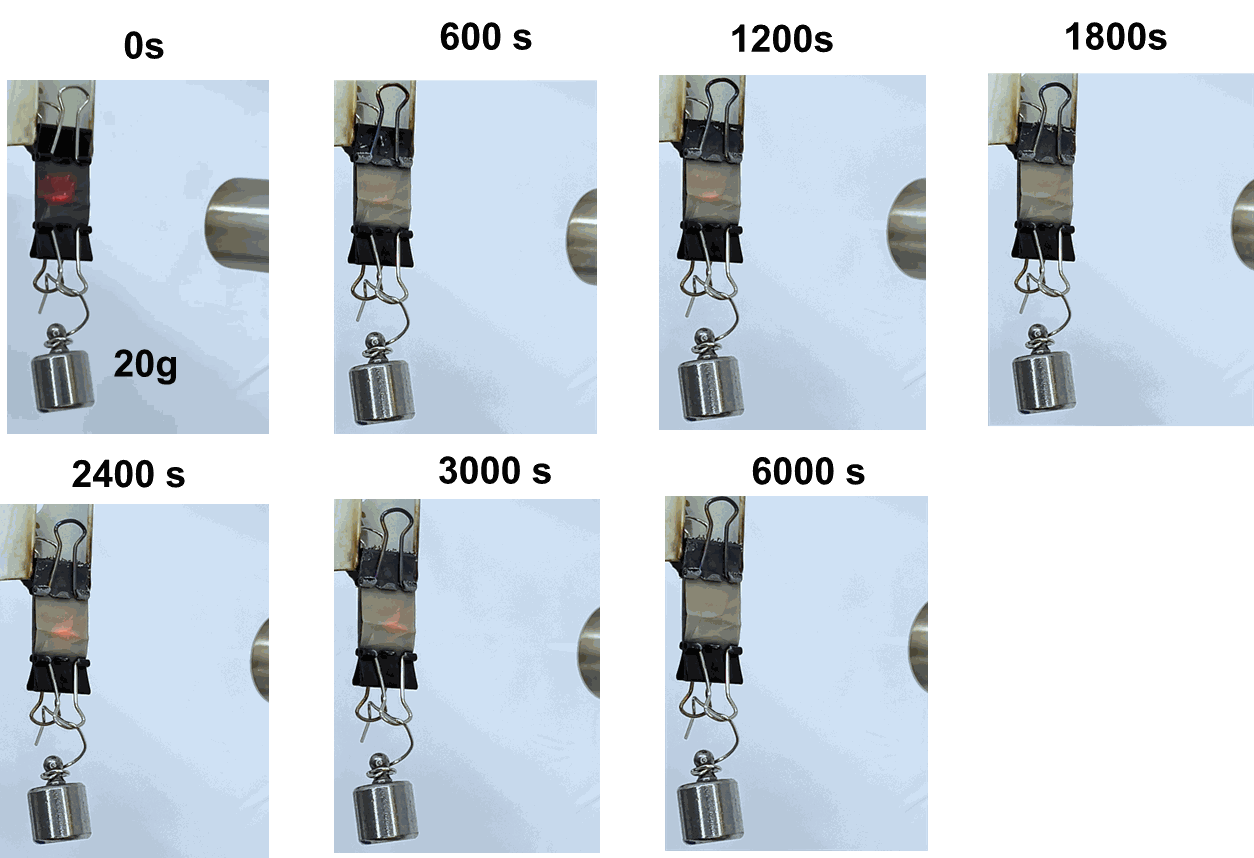


**Fig. S58** Photos of the TiC-SiC ultrafine fibrous membrane (length: 3 cm, width: 1.5 cm, and weight: 0.0140 g) pulled by a weight of 20 g under continuous heating by the butane flame


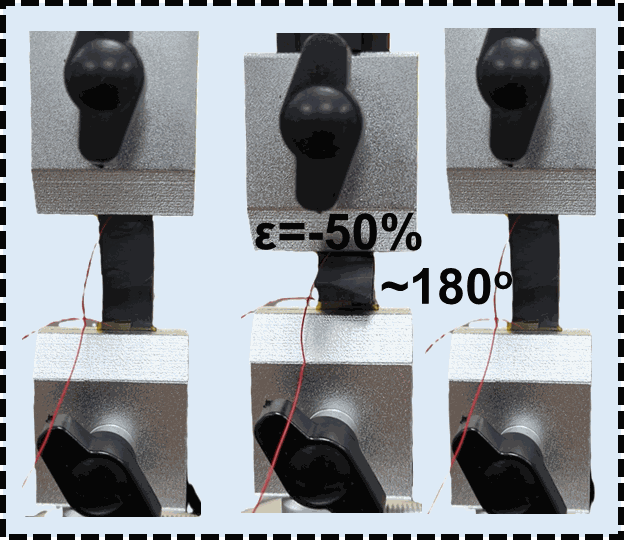


**Fig. S59** Optical photos of the TiC-SiC fibrous membrane under bending process


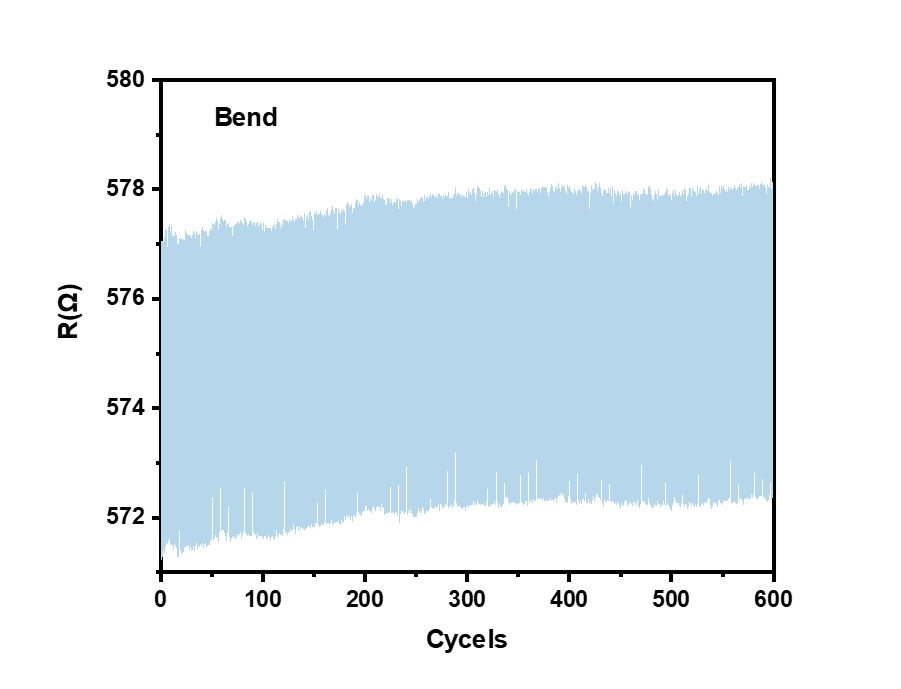


**Fig. S60** Electric resistance–cycle responses of the TiC-SiC fibrous membrane under bending and relaxing


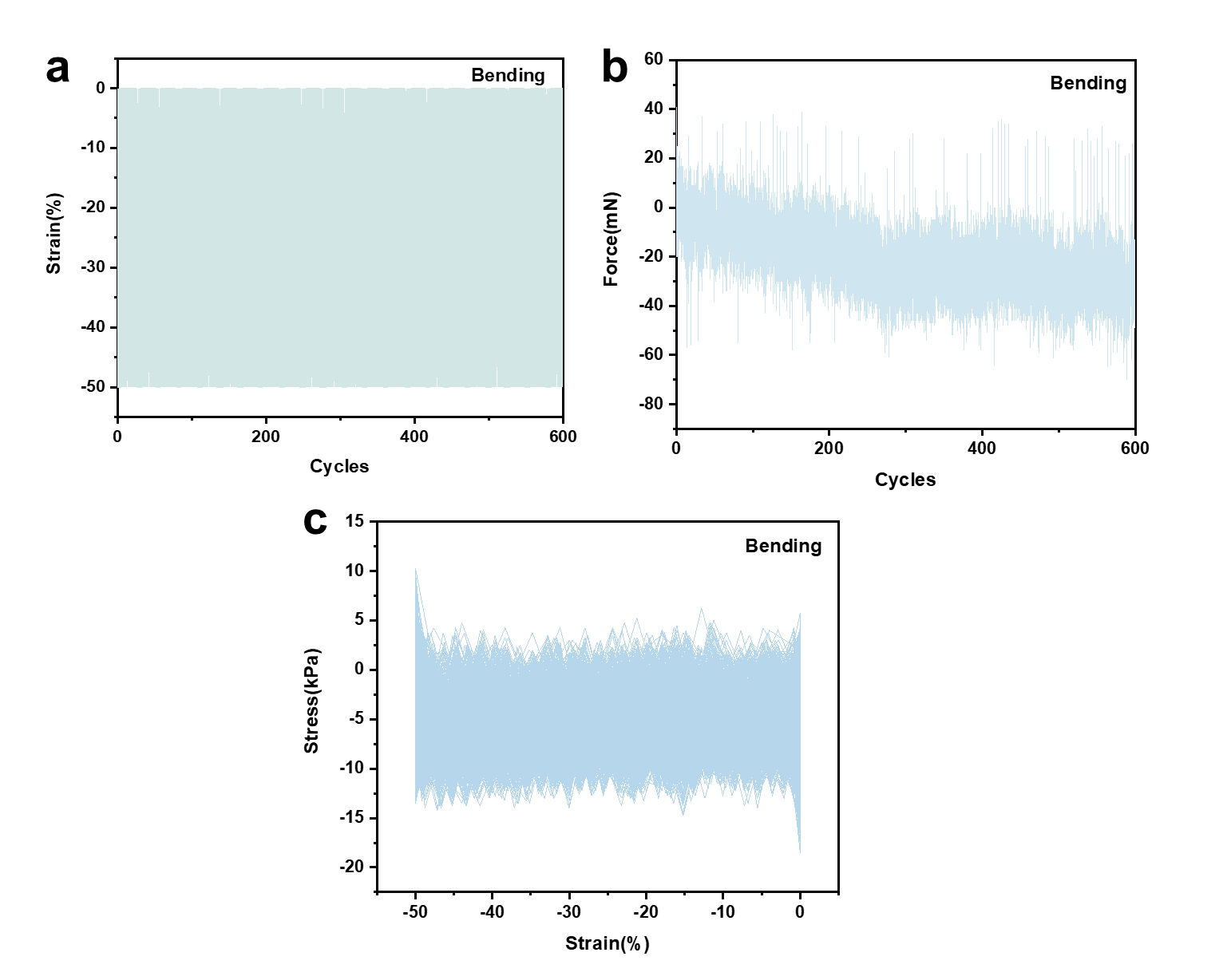


**Fig. S61** The mechanical properties of the TiC-SiC fibrous membrane during bending process


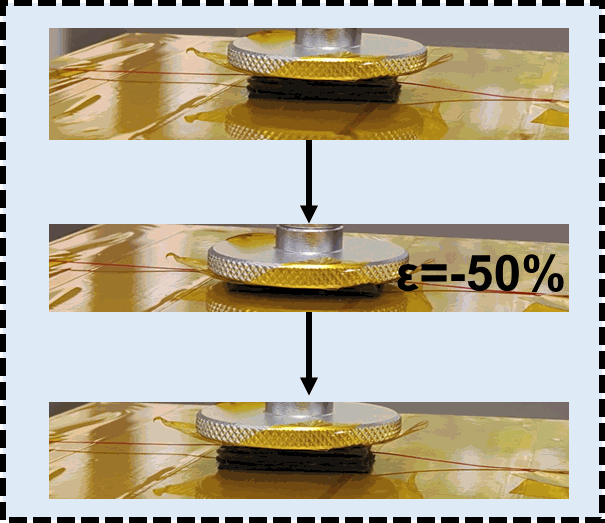


**Fig. S62** Optical photos of the stacked TiC-SiC fibrous membranes under compression process


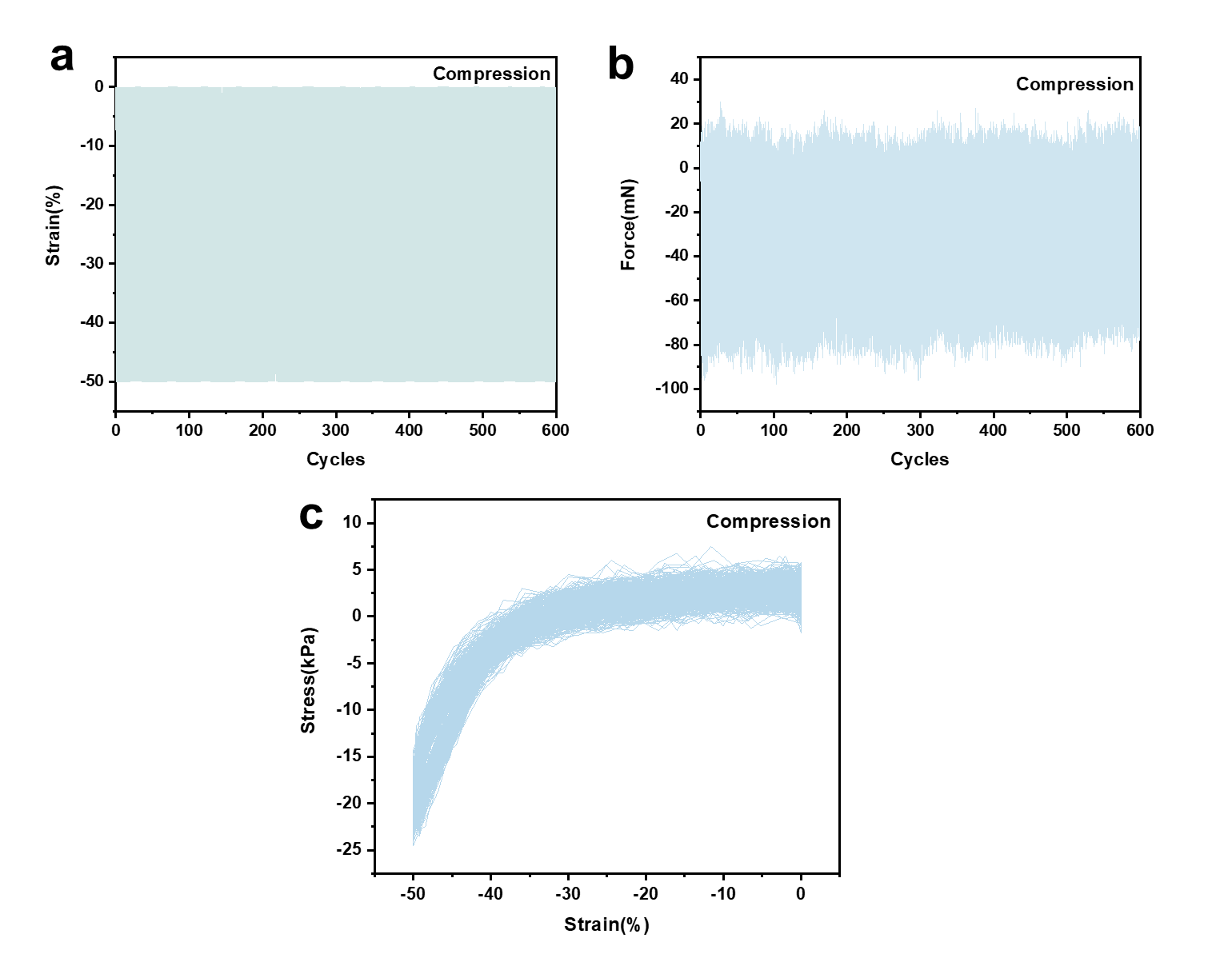


**Fig. S63** The mechanical properties of the TiC-SiC fibrous membrane during compression process


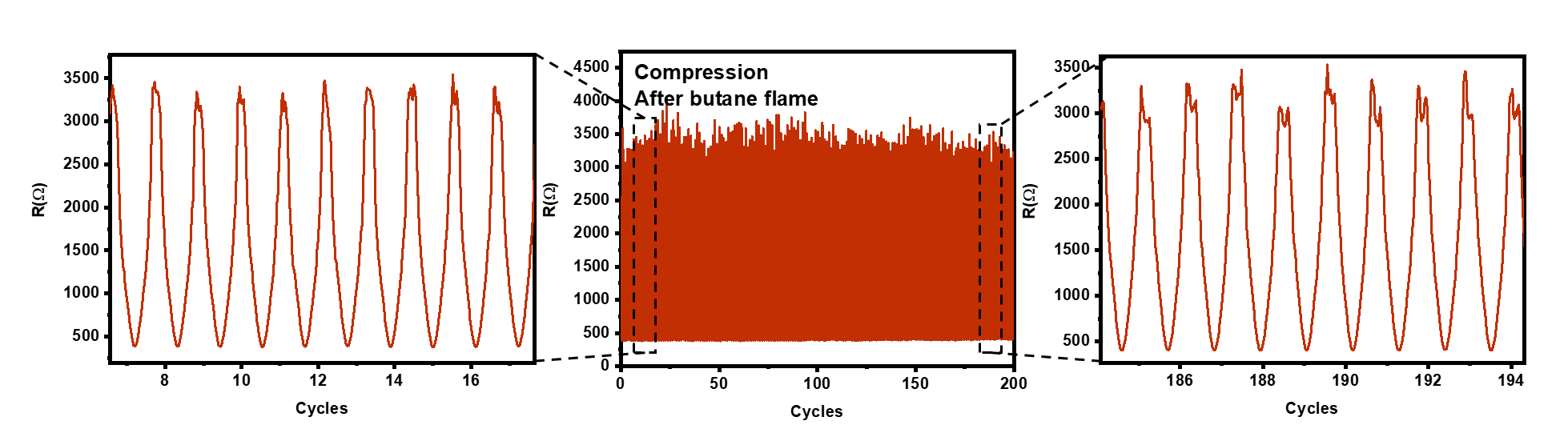


**Fig. S64** Electric resistance–cycle responses of the stacked TiC-SiC fibrous membrane (after butane flame ablation for 60 s) under compression and relaxing


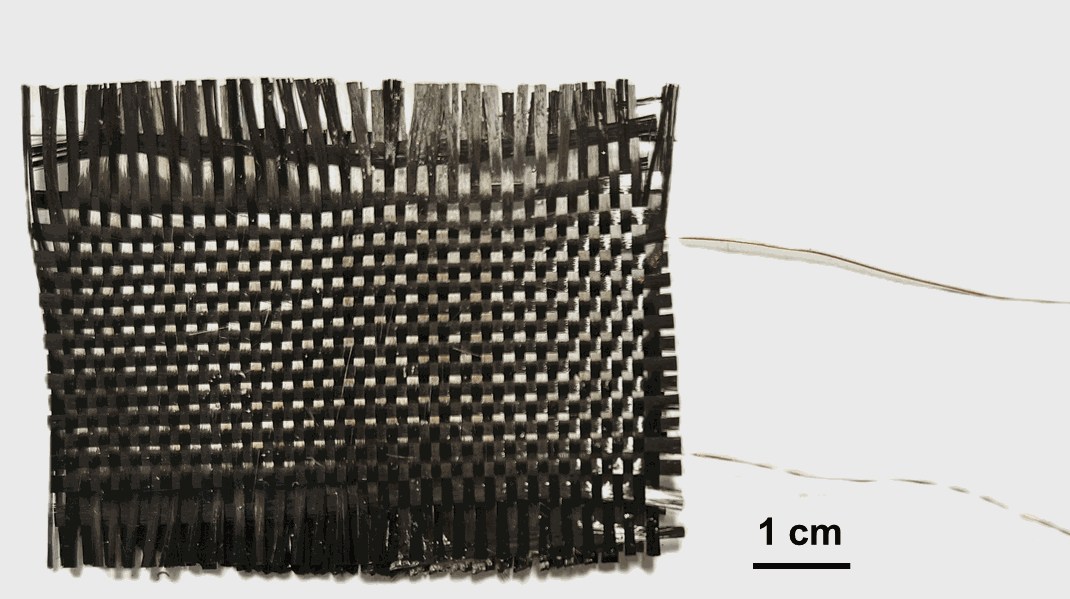


**Fig. S65** Optical photos of sensor devices assembled by the TiC-SiC fibrous membranes

**Table S7** Detailed data list of radar chart

| Material | Tensile strength | Working temperature in Ar (°C) | Working time in Ar | Oxidation resistance | Pressure sensing performance | Refs. |
| --- | --- | --- | --- | --- | --- | --- |
| Montmorillonite@ZrO_2_-SiO_2_ | 1.83 MPa | 1000 | ~10 min | ~1000 ^o^C | / | [S5] |
| SiC | / | 1800 | 60min | 1400 ^o^C | 100 cycles in room temperature | [S12] |
| SiC@SiO_2_ | / | 600 | ~10min | 700 ^o^C | 1000 cycles at room temperature (ɛ=30%) | [S13] |
| SCNFM | 2.16 MPa | 700 | 120 min | / | / | [S14] |
| ZSNFA | ~1.8 MPa | 1100 | ~15 min | ~1300 | 1000 cycles  (800 ℃) | [S15] |
| TiO_2_ | / | 370 | ~1 min | 1300 | 10000 cycles (room temperature)  5 cycles (370 ℃) | [S16] |
| This work | 2.1MPa | 1800 | 300 min | 1200:60 min  1300: 60min | 600 cycles (room temperature, ɛ=50%)  600 cycles (after butane blame, ɛ=50%) |  |

It was noted that:

1.The stability of Submicron SiC fibers in the air was judged based on the fact that the mass increased 5.05 wt% according to TG.

2.The stability in the air of SiC@SiO_2_ nanofiber aerogel was based on alcohol lamp flame reported in the literature.

3. SCNFM refers to SiO_2_-carbon nanofiber membranes. The working temperature and time were selected based on the preparation temperature and time of membrane reported in the literature.

4. ZSNFA refers to ZrO_2_-SiO_2_ nanofiber aerogel.

5. In terms of pressure sensing performance, we comprehensively considered the combined factors of the number of cycles and the working temperature.

**Supplementary References**

1. W. Zhao, X. Wang, M. Liu, Fabrication of *in situ* carbon coatings and their influence on mechanical properties of SiC fibers. Ceram. Int. **51**(8), 10402–10414 (2025). https://doi.org/10.1016/j.ceramint.2024.12.473
2. M. Sreemany, T.B. Ghosh, B.C. Pai, M. Chakraborty, XPS studies on the oxidation behavior of SiC particles. Mater. Res. Bull. **33**(2), 189–198 (1998). https://doi.org/10.1016/S0025-5408(97)00222-5
3. J.E. Oghenevweta, D. Wexler, A. Calka, Study of reaction sequences during MSR synthesis of TiC by controlled ball milling of titanium and graphite. Mater. Charact. **140**, 299–311 (2018). https://doi.org/10.1016/j.matchar.2018.04.005
4. L. Pang, P. Xiao, Z. Li, H. Luo, J. Zheng et al., Long-range uniform SiC(x)O(y) beaded carbon fibers for efficient microwave absorption. ACS Appl. Mater. Interfaces **15**(25), 30815–30825 (2023). https://doi.org/10.1021/acsami.3c05029
5. X. Mao, J. Hong, Y.-X. Wu, Q. Zhang, J. Liu et al., An efficient strategy for reinforcing flexible ceramic membranes. Nano Lett. **21**(22), 9419–9425 (2021). https://doi.org/10.1021/acs.nanolett.1c02657
6. Y. Zhang, S. Liu, J. Yan, X. Zhang, S. Xia et al., Superior flexibility in oxide ceramic crystal nanofibers. Adv. Mater. **33**(44), 2105011 (2021). https://doi.org/10.1002/adma.202105011
7. Z. Xu, Y. Liu, Q. Xin, J. Dai, J. Yu et al., Ceramic meta-aerogel with thermal superinsulation up to 1700 °C constructed by self-crosslinked nanofibrous network *via* reaction electrospinning. Adv. Mater. **36**(32), 2401299 (2024). https://doi.org/10.1002/adma.202401299
8. Y. Wang, W. Qin, Z. Chen, Z. Deng, D. Ma et al., Fine-grain high-performance densified oxide fibers produced by open ultrafast high-temperature sintering. Adv. Mater. **36**(48), 2412139 (2024). https://doi.org/10.1002/adma.202412139
9. X. Wang, Z. Zhu, Z. Guo, C. Mi, Z. Huang et al., Thermally insulating and wave-transparent magnesium aluminum spinel nanofibers applied from–196 to 1600 °C. Chem. Eng. J. **502**, 157617 (2024). https://doi.org/10.1016/j.cej.2024.157617
10. X. Zhang, B. Wang, N. Wu, C. Han, C. Wu et al., Flexible and thermal-stable SiZrOC nanofiber membranes with low thermal conductivity at high-temperature. J. Eur. Ceram. Soc. **40**(5), 1877–1885 (2020). https://doi.org/10.1016/j.jeurceramsoc.2020.01.037
11. J. Chen, Y. Zhang, D. Yan, Y. Gou, Flexible ultrafine nearly stoichiometric polycrystalline SiC fibers with excellent oxidation resistance and superior thermal stability up to 1900 °C. J. Eur. Ceram. Soc. **42**(5), 1938–1946 (2022). https://doi.org/10.1016/j.jeurceramsoc.2021.12.049
12. W. Kang, Y. Shen, T. Yang, Z. Zhao, Y. Gou, Multifunctional submicron SiC fibers for extreme environments: superior electromagnetic absorption and high-temperature performance. Adv. Funct. Mater. **35**(7), 2415432 (2025). https://doi.org/10.1002/adfm.202415432
13. L. Song, F. Zhang, Y. Chen, L. Guan, Y. Zhu et al., Multifunctional SiC@SiO_2_ nanofiber aerogel with ultrabroadband electromagnetic wave absorption. Nanomicro Lett. **14**(1), 152 (2022). https://doi.org/10.1007/s40820-022-00905-6
14. H. Zhao, Z. Jian, J. Zhang, Y. Du, Z. Tang et al., Controllable preparation of carbon nanofiber membranes for enhanced flexibility and permeability. Carbon **229**, 119496 (2024). https://doi.org/10.1016/j.carbon.2024.119496
15. W. Xiao, L. Lu, Z. Xu, Y. Huang, Q. Zhuang et al., A superelastic ceramic aerogel for flexible pressure sensor in harsh environment. Compos. Part B Eng. **292**, 112110 (2025). https://doi.org/10.1016/j.compositesb.2024.112110
16. M. Fu, J. Zhang, Y. Jin, Y. Zhao, S. Huang et al., A highly sensitive, reliable, and high-temperature-resistant flexible pressure sensor based on ceramic nanofibers. Adv. Sci. **7**(17), 2000258 (2020). https://doi.org/10.1002/advs.202000258
